# Supplementary material for: Combined physical and pharmacological anabolic osteoporosis therapies increase bone response and mechanoregulation in female mice
Source: Nat Commun. 2026 Mar 10;17:3759. doi: 10.1038/s41467-026-70309-2 (PMC13106859; doi:10.1038/s41467-026-70309-2)
Supplement: Supplementary file 1 — Supplementary Information [file 41467_2026_70309_MOESM1_ESM.pdf]

Supplementary material

Supplementary Dataset S.1: Color encoding for significant differences to a specific group. Significances are depicted with a symbol in the color of the corresponding group using two-way ANOVA for the shown groups followed by Tukey’s post-hoc test (p<0.05: **o**: to monotreatment, ●: to combined treatment, ooooo●●●: to VEH 0N, to VEH 8N, to BIS 0N, to PTH 0N, to ASC 0N, to BIS 8N, to PTH 8N, to ASC 8N).

o	o	o	o	o	●	●	●
VEH 0N	VEH 8N	BIS 0N	PTH 0N	SclAB 0N	BIS 8N	PTH 8N	SclAB 8N

Supplementary Dataset S.2: Static morphometric and mechanical parameters of the trabecular compartment of non-loaded (0N) and loaded (8N) 6th caudal vertebrae at weeks 15, 20, 22, and 24 (w15, w20, w22, w24). Values are presented as group means ± standard error, Δ represents the %-change from week 20 to 24 (Δw20-24). Two-way ANOVA over time for the shown groups followed by Tukey’s post-hoc test was used on Δw20-24. ‘x’ denotes the reference group; asterisks (*, **, ***) indicate groups that are significantly different from 'x'. Significance is indicated as: *p<0.05, **p<0.01, ***p<0.001. Synergy (S) or antagonism (I) between treatment and mechanical loading was assessed using the interaction term “treatment:loading” from a fitted linear model applied to Δw20-24.

Readout	N	w15	**w20 (baseline)**	w22	w24	**Δw20-24 [%]**	o	o	o	o	o	•	•	•	Synergy
**VEH (0N: n=25; 8N: n=26)**															
BV/TV [%]	0N	18.900 ± 0.444	14.319 ± 0.416	12.801 ± 0.418	12.832 ± 0.372	-10.051 ± 1.525	x	*** <0.001	*** <0.001	*** <0.001	*** <0.001	*** <0.001	*** <0.001	*** <0.001	-
	8N	19.650 ± 0.590	14.692 ± 0.306	15.761 ± 0.319	17.206 ± 0.343	17.652 ± 2.166	*** <0.001	x	0.996	1.000	0.938	0.204	*** <0.001	*** <0.001	-
Tb.Th [µm]	0N	74.916 ± 0.984	65.004 ± 0.691	63.889 ± 0.802	65.257 ± 0.724	0.471 ± 0.877	x	*** <0.001	0.304	** 0.002	*** <0.001	*** <0.001	*** <0.001	*** <0.001	-
	8N	76.215 ± 1.112	66.913 ± 0.735	73.023 ± 0.688	80.531 ± 0.909	20.606 ± 1.620	*** <0.001	x	*** <0.001	*** <0.001	0.957	0.996	*** <0.001	*** <0.001	-
Tb.N [1/mm]	0N	3.054 ± 0.066	2.990 ± 0.066	2.819 ± 0.070	2.727 ± 0.064	-8.777 ± 0.803	x	0.985	*** <0.001	0.384	0.823	*** <0.001	0.487	1.000	-
	8N	3.128 ± 0.065	2.975 ± 0.045	2.814 ± 0.049	2.736 ± 0.048	-8.071 ± 0.663	0.985	x	*** <0.001	0.817	0.395	*** <0.001	0.880	0.998	-
Conn.D [1/mm^3]	0N	59.813 ± 2.222	62.528 ± 2.213	55.359 ± 2.327	48.863 ± 1.797	-21.236 ± 2.242	x	0.187	*** <0.001	*** <0.001	* 0.019	0.955	0.082	*** <0.001	-
	8N	62.461 ± 3.573	61.758 ± 1.815	51.572 ± 2.309	43.168 ± 1.661	-29.454 ± 2.523	0.187	x	*** <0.001	*** <0.001	0.774	0.068	0.965	*** <0.001	-
Strength [N]	0N	35.872 ± 0.792	24.467 ± 0.830	23.863 ± 0.665	25.511 ± 0.597	6.010 ± 2.751	x	*** <0.001	*** <0.001	*** <0.001	*** <0.001	*** <0.001	*** <0.001	*** <0.001	-
	8N	35.551 ± 1.177	26.061 ± 0.723	30.414 ± 0.595	34.597 ± 0.538	34.657 ± 3.451	*** <0.001	x	0.972	1.000	0.848	0.787	*** <0.001	*** <0.001	-
SED [kPa]	0N	1.328 ± 0.045	2.369 ± 0.167	2.397 ± 0.138	2.152 ± 0.087	-5.127 ± 3.305	x	*** <0.001	*** <0.001	*** <0.001	*** <0.001	*** <0.001	*** <0.001	*** <0.001	-
	8N	5.589 ± 0.347	8.298 ± 0.345	6.483 ± 0.184	5.287 ± 0.124	-34.240 ± 2.278	*** <0.001	x	0.991	0.999	0.908	0.156	*** <0.001	*** <0.001	-
eff [µε]	0N	386.583 ± 6.055	486.519 ± 13.443	490.780 ± 11.852	471.776 ± 8.528	-2.334 ± 1.387	x	*** <0.001	*** <0.001	*** <0.001	*** <0.001	*** <0.001	*** <0.001	*** <0.001	-
	8N	777.540 ± 14.223	926.800 ± 17.599	836.932 ± 10.786	767.422 ± 8.612	-16.700 ± 1.265	*** <0.001	x	0.930	0.981	0.806	* 0.047	*** <0.001	*** <0.001	-

**BIS (0N: n=9; 8N: n=9)**															
BV/TV [%]	0N	17.249 ± 0.844	13.119 ± 0.381	13.965 ± 0.209	14.942 ± 0.224	14.448 ± 2.834	*** <0.001	0.996	x	0.972	0.773	0.147	*** <0.001	*** <0.001	-
	8N	18.046 ± 0.800	14.315 ± 0.538	15.827 ± 0.506	18.287 ± 0.564	28.040 ± 1.483	*** <0.001	0.204	0.147	0.693	0.961	x	*** <0.001	*** <0.001	**I* 0.029**
Tb.Th [µm]	0N	68.818 ± 1.542	63.445 ± 0.775	64.994 ± 0.933	66.507 ± 1.143	4.832 ± 1.334	0.304	*** <0.001	x	0.873	*** <0.001	*** <0.001	*** <0.001	*** <0.001	-
	8N	72.772 ± 1.571	65.685 ± 1.408	70.857 ± 1.423	78.318 ± 1.598	19.271 ± 0.832	*** <0.001	0.996	*** <0.001	* 0.038	0.808	x	*** <0.001	*** <0.001	0.228
Tb.N [1/mm]	0N	3.049 ± 0.091	2.900 ± 0.087	2.890 ± 0.082	2.929 ± 0.079	1.092 ± 0.968	*** <0.001	*** <0.001	x	*** <0.001	*** <0.001	0.563	*** <0.001	*** <0.001	-
	8N	3.002 ± 0.075	2.928 ± 0.077	2.883 ± 0.086	2.887 ± 0.070	-1.365 ± 0.722	*** <0.001	*** <0.001	0.563	** 0.001	*** <0.001	x	** 0.001	*** <0.001	0.059
Conn.D [1/mm^3]	0N	73.848 ± 5.318	65.494 ± 3.108	66.346 ± 2.951	65.816 ± 3.452	1.111 ± 5.102	*** <0.001	*** <0.001	x	0.999	*** <0.001	* 0.037	*** <0.001	*** <0.001	-
	8N	61.471 ± 4.498	66.525 ± 4.171	62.080 ± 4.280	54.486 ± 3.530	-17.793 ± 2.654	0.955	0.068	* 0.037	0.146	** 0.006	x	* 0.025	*** <0.001	0.115
Strength [N]	0N	33.850 ± 1.639	24.078 ± 1.059	28.051 ± 0.608	30.169 ± 0.503	26.707 ± 4.422	*** <0.001	0.972	x	0.957	0.485	0.421	*** <0.001	*** <0.001	-
	8N	35.956 ± 1.330	25.134 ± 0.774	30.766 ± 0.844	35.722 ± 0.938	42.376 ± 1.905	*** <0.001	0.787	0.421	0.966	1.000	x	*** <0.001	*** <0.001	0.195
SED [kPa]	0N	1.444 ± 0.107	2.283 ± 0.154	1.778 ± 0.060	1.590 ± 0.055	-28.601 ± 3.570	*** <0.001	0.991	x	1.000	0.67	0.093	*** <0.001	*** <0.001	-
	8N	5.121 ± 0.353	8.256 ± 0.364	5.893 ± 0.193	4.661 ± 0.147	-43.254 ± 1.107	*** <0.001	0.156	0.093	0.139	0.957	x	0.222	*** <0.001	**I* 0.031**
eff [µε]	0N	398.883 ± 14.998	478.677 ± 14.787	433.711 ± 7.577	413.977 ± 7.597	-13.147 ± 1.754	*** <0.001	0.930	x	1.000	0.342	* 0.012	*** <0.001	*** <0.001	-
	8N	752.617 ± 22.149	915.854 ± 15.201	790.326 ± 9.229	712.192 ± 8.389	-22.168 ± 0.729	*** <0.001	* 0.047	* 0.012	* 0.020	0.903	x	0.054	*** <0.001	0.121
**PTH (0N: n=10; 8N: n=9)**															
BV/TV [%]	0N	23.586 ± 0.775	15.701 ± 0.685	16.515 ± 0.532	19.034 ± 0.585	22.126 ± 3.170	*** <0.001	1.000	0.972	x	0.999	0.693	*** <0.001	*** <0.001	-
	8N	24.396 ± 1.283	17.112 ± 0.514	20.879 ± 0.331	24.870 ± 0.672	46.320 ± 5.608	*** <0.001	*** <0.001	*** <0.001	*** <0.001	*** <0.001	*** <0.001	x	0.262	0.592
Tb.Th [µm]	0N	81.480 ± 1.464	64.539 ± 1.290	65.570 ± 0.969	71.904 ± 1.165	11.579 ± 1.499	** 0.002	*** <0.001	0.873	x	*** <0.001	* 0.038	*** <0.001	*** <0.001	-
	8N	81.164 ± 3.793	65.286 ± 1.728	76.065 ± 1.834	86.769 ± 2.229	33.334 ± 3.763	*** <0.001	*** <0.001	*** <0.001	*** <0.001	* 0.010	*** <0.001	x	*** <0.001	0.671
Tb.N [1/mm]	0N	3.314 ± 0.067	3.182 ± 0.049	3.027 ± 0.051	2.981 ± 0.044	-6.285 ± 0.925	0.384	0.817	*** <0.001	x	0.059	** 0.001	1.000	0.673	-
	8N	3.546 ± 0.092	3.386 ± 0.077	3.220 ± 0.084	3.172 ± 0.077	-6.331 ± 0.842	0.487	0.880	*** <0.001	1.000	0.086	** 0.001	x	0.739	0.605
Conn.D [1/mm^3]	0N	53.684 ± 2.677	61.894 ± 2.880	63.813 ± 4.428	57.276 ± 4.012	-6.361 ± 6.479	*** <0.001	*** <0.001	0.999	x	*** <0.001	0.146	*** <0.001	*** <0.001	-
	8N	63.366 ± 5.082	75.111 ± 3.933	56.492 ± 3.811	53.270 ± 3.905	-28.998 ± 3.673	0.082	0.965	*** <0.001	*** <0.001	1.000	* 0.025	x	0.100	**I* 0.032**
Strength [N]	0N	40.605 ± 1.161	24.955 ± 1.130	28.662 ± 0.647	34.113 ± 0.598	38.630 ± 4.967	*** <0.001	1.000	0.957	x	0.98	0.966	*** <0.001	*** <0.001	-
	8N	42.442 ± 1.989	27.409 ± 1.160	37.233 ± 0.684	46.164 ± 0.789	70.600 ± 7.118	*** <0.001	*** <0.001	*** <0.001	*** <0.001	*** <0.001	*** <0.001	x	* 0.021	0.692
SED [kPa]	0N	1.136 ± 0.072	2.358 ± 0.185	1.938 ± 0.080	1.478 ± 0.056	-35.309 ± 3.503	*** <0.001	0.999	1.000	x	0.787	0.139	*** <0.001	*** <0.001	-
	8N	4.250 ± 0.374	7.958 ± 0.515	4.920 ± 0.159	3.531 ± 0.112	-54.392 ± 2.848	*** <0.001	*** <0.001	*** <0.001	*** <0.001	* 0.011	0.222	x	0.557	0.126
eff [µε]	0N	363.814 ± 10.526	497.278 ± 16.297	464.109 ± 9.004	411.071 ± 7.738	-16.890 ± 1.919	*** <0.001	0.981	1.000	x	0.463	* 0.020	*** <0.001	*** <0.001	-
	8N	699.888 ± 29.012	921.744 ± 25.290	752.274 ± 12.408	646.306 ± 11.010	-29.526 ± 2.025	*** <0.001	*** <0.001	*** <0.001	*** <0.001	*** <0.001	0.054	x	0.106	0.620
**SclAB (0N: n=9; 8N: n=8)**															
BV/TV [%]	0N	23.456 ± 1.169	15.295 ± 0.805	16.158 ± 0.758	19.012 ± 0.853	25.329 ± 4.223	*** <0.001	0.938	0.773	0.999	x	0.961	*** <0.001	*** <0.001	-
	8N	23.176 ± 0.994	15.116 ± 0.518	18.935 ± 0.740	23.922 ± 0.834	59.408 ± 7.448	*** <0.001	*** <0.001	*** <0.001	*** <0.001	*** <0.001	*** <0.001	0.262	x	0.311

Tb.Th [μm]	0N	79.495 ± 2.283	63.984 ± 1.509	69.422 ± 1.345	79.953 ± 1.374	25.268 ± 2.307	*** <0.001	0.957	*** <0.001	*** <0.001	x	0.808	* 0.010	*** <0.001	-
	8N	80.377 ± 2.471	62.352 ± 2.108	76.745 ± 2.556	93.350 ± 4.963	49.949 ± 6.862	*** <0.001	*** <0.001	*** <0.001	*** <0.001	*** <0.001	*** <0.001	*** <0.001	x	0.297
Tb.N [1/mm]	0N	3.328 ± 0.092	3.113 ± 0.090	2.906 ± 0.080	2.787 ± 0.074	-10.424 ± 0.900	0.823	0.395	*** <0.001	0.059	x	*** <0.001	0.086	0.944	-
	8N	3.366 ± 0.084	3.286 ± 0.112	3.081 ± 0.121	3.017 ± 0.122	-8.310 ± 0.632	1.000	0.998	*** <0.001	0.673	0.944	*** <0.001	0.739	x	0.467
Conn.D [1/mm^3]	0N	68.842 ± 4.411	68.475 ± 5.835	52.715 ± 3.706	43.315 ± 2.051	-35.023 ± 2.766	* 0.019	0.774	*** <0.001	*** <0.001	x	** 0.006	1.000	0.249	-
	8N	58.353 ± 4.786	79.184 ± 11.397	53.348 ± 8.541	43.297 ± 6.892	-45.407 ± 2.772	*** <0.001	*** <0.001	*** <0.001	*** <0.001	0.249	*** <0.001	0.100	x	0.733
Strength [N]	0N	39.602 ± 1.627	24.416 ± 0.906	28.509 ± 1.228	35.787 ± 1.268	47.028 ± 3.567	*** <0.001	0.848	0.485	0.980	x	1.000	*** <0.001	*** <0.001	-
	8N	41.085 ± 1.952	23.236 ± 1.413	33.566 ± 1.230	44.257 ± 1.330	95.209 ± 12.862	*** <0.001	*** <0.001	*** <0.001	*** <0.001	*** <0.001	*** <0.001	* 0.021	x	**S* 0.045**
SED [kPa]	0N	1.161 ± 0.066	2.378 ± 0.173	1.908 ± 0.124	1.318 ± 0.070	-43.770 ± 2.342	*** <0.001	0.908	0.670	0.787	x	0.957	* 0.011	*** <0.001	-
	8N	4.492 ± 0.302	10.492 ± 1.156	5.623 ± 0.248	3.696 ± 0.187	-62.794 ± 3.192	*** <0.001	*** <0.001	*** <0.001	*** <0.001	*** <0.001	*** <0.001	0.557	x	0.440
eff [με]	0N	366.189 ± 9.662	497.919 ± 17.193	454.488 ± 13.976	385.734 ± 10.069	-22.275 ± 1.500	*** <0.001	0.806	0.342	0.463	x	0.903	*** <0.001	*** <0.001	-
	8N	722.750 ± 22.926	1030.171 ± 40.492	790.286 ± 17.042	654.367 ± 17.076	-35.938 ± 2.566	*** <0.001	*** <0.001	*** <0.001	*** <0.001	*** <0.001	*** <0.001	0.106	x	0.859

Supplementary Dataset S.3: Absolute values and %-changes in bone volume fraction (BV/TV) of the three vehicle groups of the four experiments (see Methods). The animals in Experiment 3 (SclAB) were supplied by a different provider; see Methods section “Study design”. The VEH 0N groups received sham loading, the VEH 8N groups cyclic mechanical loading according to an established loading protocol[1] in the sixth caudal vertebra between week 20 and 24. Values are presented as group means ± standard error in weeks 15, 20, 22, and 24 (w15, w20, w22, w24), Δ represents the %-change from week 20 to 24 (Δw20-24). One-way ANOVA for the shown vehicle groups followed by Tukey’s post-hoc test was used to calculate significant differences between groups. *p<0.05, **p<0.01, ***p<0.001.

BV/TV [%]	w15	**w20 (baseline)**	w22	w24	Δw20-24 [%]
**Analysis 1: VEH 0N (n = 25)**					
Experiment 1	17.398 ± 0.489	13.172 ± 0.741	11.467 ± 0.560	11.247 ± 0.524	-13.840 ± 2.731
Experiment 2	18.854 ± 0.650	14.204 ± 0.378	13.065 ± 0.310	13.387 ± 0.293	-5.606 ± 1.390
Experiment 3	20.636 ± 0.765	15.724 ± 0.716	14.036 ± 0.928	14.060 ± 0.615	-10.232 ± 2.875
P-value: 1 vs. 2; 2 vs. 3; 1 vs. 3	0.861; 0.673; ***0.014**	0.987; 0.849; 0.130	0.773; 0.994; 0.124	0.350; 1.000; 0.060	0.064;0.408; 0.557
**Analysis 2: VEH 8N (n = 26)**					
Experiment 1	16.998 ± 0.884	13.811 ± 0.733	14.369 ± 0.680	15.421 ± 0.635	12.388 ± 3.810
Experiment 2	18.809 ± 0.699	14.714 ± 0.527	15.776 ± 0.431	17.231 ± 0.321	17.842 ± 3.044
Experiment 3	22.179 ± 0.573	15.288 ± 0.324	16.721 ± 0.345	18.434 ± 0.405	21.166 ± 4.020
P-value: 1 vs. 2; 2 vs. 3; 1 vs. 3	0.534; **0.001; *<0.001**	0.992; 1.000; 0.7551	0.825; 0.976; 0.114	0.493; 0.878; ****0.010**	0.588; 0.786; 0.252

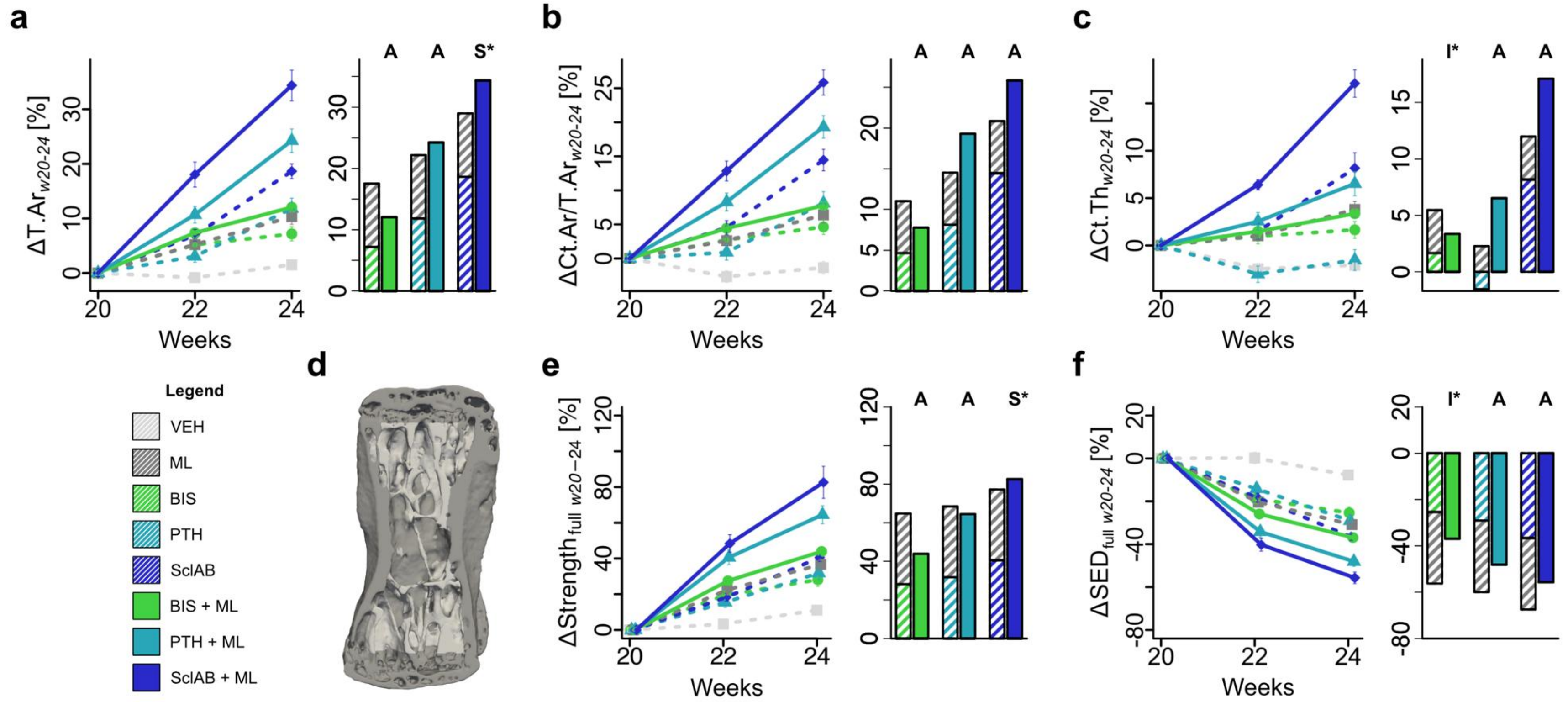

Supplementary Figure S.1: Mechanical loading increases the cortical effects of anabolic and anti-catabolic treatments, contributing to increased predicted strength. (a-f) Changes from baseline. Data are presented as mean ± standard error. Each data point represents one animal (biological replicate; VEH: n=25, ML: n=26, BIS 0N: n=9, BIS 8N: n=9, PTH 0N: n=10, PTH 8N: n=9, SclAB 0N: n=9, SclAB 8N: n=8) i **(a)** total area (ΔT.Ar) **(b)** cortical area per total area (ΔCt.Ar/T.Ar) **(c)** cortical thickness (ΔCt.Th). **(d)** Visual representation of a representative cortical shell of a VEH 0N animal at week 20 **(e)** full predicted strength including both trabecular and cortical compartments (Δfull Strength), and **(f)** full strain energy density (Δfull SED). Bars show group means of sum of single vs. combined treatment indicating: antagonistic (**I**), additive (A) or synergistic (S) effects determined via the interaction term “treatment:loading” from a linear model on the %-change from week 20 to 24. Source data are provided as a Source Data file.

Supplementary Dataset S.4: Cortical morphometric and full mechanical parameters of the non-loaded (0N) and loaded (8N) 6th caudal vertebrae. Values are given as group means ± standard error, Δ represents the %-change from week 20 to 24 (Δw20-24). ‘full’ denotes that both trabecular and cortical compartments were included. Two-way ANOVA over time for the shown animal groups followed by Tukey’s post-hoc test was used on Δw20-24. ‘x’ denotes the reference group; asterisks (*, **, ***) indicate groups that are significantly different from 'x'. Significance is indicated as: *p<0.05, **p<0.01, ***p<0.001. Synergy (S) or antagonism (I) between treatment and mechanical loading was assessed using the interaction term “treatment:loading” from a fitted linear model applied to Δw20-24.

Readout	N	w15	**w20 (baseline)**	w22	w24	**Δw20-24 [%]**	○	○	○	○	○	●	●	●	Synergy
**VEH (0N: n=25; 8N: n=26)**															
Tt.Ar [mm^2]	0N	0.782 ± 0.011	0.690 ± 0.013	0.683 ± 0.013	0.699 ± 0.011	1.512 ± 0.906	x	*** <0.001	*** <0.001	*** <0.001	*** <0.001	*** <0.001	*** <0.001	*** <0.001	-
	8N	0.784 ± 0.016	0.719 ± 0.015	0.754 ± 0.012	0.789 ± 0.010	10.352 ± 1.435	*** <0.001	x	0.941	1.000	** 0.008	0.861	*** <0.001	*** <0.001	-
Ct.Ar/Tt.Ar [%]	0N	39.691 ± 0.437	33.846 ± 0.544	32.899 ± 0.507	33.319 ± 0.456	-1.350 ± 0.923	x	*** <0.001	*** <0.001	*** <0.001	*** <0.001	*** <0.001	*** <0.001	*** <0.001	-
	8N	39.412 ± 0.669	34.560 ± 0.528	35.412 ± 0.444	36.651 ± 0.357	6.388 ± 1.185	*** <0.001	x	0.997	1.000	*** <0.001	0.876	*** <0.001	*** <0.001	-
Ct.BS/BV [1/mm]	0N	10.299 ± 0.137	12.007 ± 0.211	12.140 ± 0.201	11.859 ± 0.159	-0.991 ± 0.925	x	*** <0.001	0.079	*** <0.001	*** <0.001	*** <0.001	*** <0.001	*** <0.001	-
	8N	10.402 ± 0.225	11.741 ± 0.200	11.319 ± 0.170	10.804 ± 0.134	-7.654 ± 1.063	*** <0.001	x	0.486	*** <0.001	* 0.011	1.000	1.000	*** <0.001	-
Ct.Th [µm]	0N	147.375 ± 1.513	138.136 ± 1.626	134.676 ± 1.629	135.171 ± 1.463	-2.078 ± 0.571	x	*** <0.001	*** <0.001	1.000	*** <0.001	*** <0.001	*** <0.001	*** <0.001	-
	8N	147.041 ± 2.279	140.147 ± 1.750	141.499 ± 1.610	145.254 ± 1.362	3.810 ± 0.829	*** <0.001	x	0.892	*** <0.001	0.076	1.000	0.181	*** <0.001	-
full Strength [N]	0N	40.461 ± 0.948	30.545 ± 0.962	31.360 ± 0.863	33.541 ± 0.715	11.041 ± 1.994	x	*** <0.001	*** <0.001	*** <0.001	*** <0.001	*** <0.001	*** <0.001	*** <0.001	-
	8N	39.320 ± 1.494	32.444 ± 0.871	39.392 ± 0.726	43.815 ± 0.594	36.657 ± 2.946	*** <0.001	x	0.595	0.530	1.000	0.519	*** <0.001	*** <0.001	-
full SED [kPa]	0N	0.926 ± 0.029	1.387 ± 0.087	1.368 ± 0.072	1.245 ± 0.049	-7.748 ± 2.248	x	*** <0.001	*** <0.001	*** <0.001	*** <0.001	*** <0.001	*** <0.001	*** <0.001	-
	8N	4.085 ± 0.372	4.929 ± 0.202	3.860 ± 0.112	3.320 ± 0.074	-30.863 ± 1.943	*** <0.001	x	0.856	0.723	0.996	0.296	*** <0.001	*** <0.001	-
full eff [µε]	0N	311.044 ± 4.443	365.964 ± 8.493	368.154 ± 8.102	355.648 ± 6.220	-2.478 ± 0.914	x	*** <0.001	*** <0.001	*** <0.001	*** <0.001	*** <0.001	*** <0.001	*** <0.001	-
	8N	629.025 ± 14.398	699.091 ± 13.042	632.674 ± 8.494	592.658 ± 6.395	-14.792 ± 1.083	*** <0.001	x	0.695	0.479	0.996	0.273	*** <0.001	*** <0.001	-
**BIS (0N: n=9; 8N: n=9)**															
Tt.Ar [mm^2]	0N	0.765 ± 0.023	0.691 ± 0.023	0.725 ± 0.018	0.738 ± 0.018	7.178 ± 1.296	*** <0.001	0.941	x	0.994	** 0.003	0.420	*** <0.001	*** <0.001	-
	8N	0.818 ± 0.020	0.727 ± 0.012	0.781 ± 0.011	0.815 ± 0.011	12.051 ± 0.579	*** <0.001	0.861	0.420	0.866	0.603	x	*** <0.001	*** <0.001	0.221
Ct.Ar/Tt.Ar [%]	0N	37.553 ± 0.705	33.151 ± 0.803	34.010 ± 0.609	34.627 ± 0.562	4.640 ± 1.113	*** <0.001	0.997	x	0.999	** 0.002	0.697	*** <0.001	*** <0.001	-
	8N	38.966 ± 0.890	33.675 ± 0.616	35.157 ± 0.579	36.279 ± 0.592	7.769 ± 0.481	*** <0.001	0.876	0.697	0.949	0.262	x	*** <0.001	*** <0.001	0.094
Ct.BS/BV [1/mm]	0N	10.714 ± 0.230	12.065 ± 0.343	11.748 ± 0.255	11.553 ± 0.218	-4.017 ± 1.282	0.079	0.486	x	*** <0.001	*** <0.001	0.925	0.561	*** <0.001	-
	8N	10.232 ± 0.286	11.630 ± 0.232	11.257 ± 0.250	10.854 ± 0.240	-6.697 ± 0.451	*** <0.001	1.000	0.925	*** <0.001	* 0.022	x	0.998	*** <0.001	0.133
Ct.Th [µm]	0N	140.764 ± 2.272	135.074 ± 2.399	136.251 ± 1.431	137.167 ± 1.609	1.655 ± 0.880	*** <0.001	0.892	x	** 0.008	* 0.014	0.958	* 0.036	*** <0.001	-

	8N	147.476 ± 2.926	139.921 ± 1.724	142.026 ± 1.953	144.650 ± 2.047	3.363 ± 0.299	*** <0.001	1.000	0.958	*** <0.001	0.261	x	0.433	*** <0.001	**I*** **0.037**
Full Strength [N]	0N	39.812 ± 1.909	30.644 ± 1.358	36.288 ± 0.857	38.908 ± 0.800	28.138 ± 3.668	*** <0.001	0.595	x	1.000	0.824	* 0.043	*** <0.001	*** <0.001	-
	8N	43.277 ± 1.518	32.238 ± 0.850	41.043 ± 0.898	46.341 ± 1.114	43.917 ± 1.870	*** <0.001	0.519	* 0.043	* 0.032	0.726	x	*** <0.001	*** <0.001	0.195
Full SED [kPa]	0N	0.984 ± 0.066	1.370 ± 0.097	1.095 ± 0.047	1.002 ± 0.038	-25.369 ± 2.774	*** <0.001	0.856	x	1.000	0.650	0.052	*** <0.001	*** <0.001	-
	8N	3.369 ± 0.187	4.774 ± 0.204	3.528 ± 0.125	3.005 ± 0.114	-36.924 ± 0.864	*** <0.001	0.296	0.052	* 0.026	0.905	x	* 0.036	*** <0.001	**I*** **0.023**
Full eff [με]	0N	321.049 ± 10.620	369.008 ± 12.270	337.400 ± 7.380	324.857 ± 6.547	-11.628 ± 1.380	*** <0.001	0.695	x	1.000	0.487	* 0.023	*** <0.001	*** <0.001	-
	8N	597.436 ± 14.782	689.779 ± 12.362	608.455 ± 9.457	566.119 ± 9.505	-17.906 ± 0.438	*** <0.001	0.273	* 0.023	** 0.009	0.893	x	** 0.002	*** <0.001	**I*** **0.020**
**PTH (0N: n=10; 8N: n=9)**															
Tt.Ar [mm^2]	0N	0.804 ± 0.013	0.662 ± 0.013	0.682 ± 0.011	0.739 ± 0.013	11.826 ± 1.881	*** <0.001	1.000	0.994	x	* 0.028	0.866	*** <0.001	*** <0.001	-
	8N	0.833 ± 0.033	0.714 ± 0.022	0.789 ± 0.018	0.885 ± 0.021	24.255 ± 2.152	*** <0.001	*** <0.001	*** <0.001	*** <0.001	0.103	*** <0.001	x	*** <0.001	0.242
Ct.Ar/Tt.Ar [%]	0N	42.335 ± 0.929	33.494 ± 0.908	33.730 ± 0.705	36.115 ± 0.691	8.132 ± 1.694	*** <0.001	1.000	0.999	x	* 0.01	0.949	*** <0.001	*** <0.001	-
	8N	42.568 ± 1.192	34.894 ± 0.967	37.733 ± 0.853	41.565 ± 0.973	19.310 ± 1.651	*** <0.001	*** <0.001	*** <0.001	*** <0.001	0.058	*** <0.001	x	** 0.005	0.198
Ct.BS/BV [1/mm]	0N	9.778 ± 0.207	12.278 ± 0.267	13.043 ± 0.248	12.840 ± 0.272	4.720 ± 1.754	*** <0.001	*** <0.001	*** <0.001	x	*** <0.001	*** <0.001	*** <0.001	*** <0.001	-
	8N	9.600 ± 0.419	12.006 ± 0.230	11.595 ± 0.169	10.972 ± 0.164	-8.509 ± 1.020	*** <0.001	1.000	0.561	*** <0.001	0.140	0.998	x	*** <0.001	**I*** **0.010**
Ct.Th [μm]	0N	154.496 ± 2.629	136.411 ± 2.520	132.155 ± 1.894	134.166 ± 2.007	-1.537 ± 1.109	1.000	*** <0.001	** 0.008	x	*** <0.001	*** <0.001	*** <0.001	*** <0.001	-
	8N	157.820 ± 5.308	141.347 ± 3.669	144.761 ± 2.963	150.293 ± 2.699	6.526 ± 1.264	*** <0.001	0.181	* 0.036	*** <0.001	1.000	0.433	x	*** <0.001	0.259
Full Strength [N]	0N	42.623 ± 0.877	30.240 ± 1.049	34.705 ± 0.752	39.581 ± 0.838	31.813 ± 3.609	*** <0.001	0.530	1.000	x	0.795	* 0.032	*** <0.001	*** <0.001	-
	8N	44.699 ± 1.587	32.872 ± 1.042	45.930 ± 0.831	53.723 ± 0.879	64.505 ± 4.979	*** <0.001	*** <0.001	*** <0.001	*** <0.001	*** <0.001	*** <0.001	x	* 0.027	0.297
Full SED [kPa]	0N	0.832 ± 0.030	1.374 ± 0.083	1.164 ± 0.044	0.959 ± 0.036	-29.069 ± 2.878	*** <0.001	0.723	1.000	x	0.516	* 0.026	*** <0.001	*** <0.001	-
	8N	3.152 ± 0.247	4.709 ± 0.265	3.058 ± 0.110	2.406 ± 0.082	-48.096 ± 2.249	*** <0.001	*** <0.001	*** <0.001	*** <0.001	*** <0.001	* 0.036	x	0.385	0.420
Full eff [με]	0N	295.311 ± 5.349	367.309 ± 9.223	345.724 ± 6.331	316.393 ± 5.798	-13.639 ± 1.487	*** <0.001	0.479	1.000	x	0.324	** 0.009	*** <0.001	*** <0.001	-
	8N	572.588 ± 21.462	684.048 ± 18.123	570.847 ± 10.029	510.037 ± 8.700	-25.182 ± 1.561	*** <0.001	*** <0.001	*** <0.001	*** <0.001	*** <0.001	** 0.002	x	0.112	0.781
**ScIAB (0N: n=9; 8N: n=8)**															
Tt.Ar [mm^2]	0N	0.782 ± 0.023	0.658 ± 0.015	0.703 ± 0.014	0.779 ± 0.011	18.645 ± 1.355	*** <0.001	** 0.008	** 0.003	* 0.028	x	0.603	0.103	*** <0.001	-
	8N	0.824 ± 0.027	0.658 ± 0.024	0.774 ± 0.022	0.881 ± 0.026	34.375 ± 2.824	*** <0.001	*** <0.001	*** <0.001	*** <0.001	*** <0.001	*** <0.001	*** <0.001	x	**S*** **0.034**
Ct.Ar/Tt.Ar [%]	0N	42.157 ± 1.502	34.044 ± 0.972	35.565 ± 0.975	38.893 ± 0.900	14.461 ± 1.580	*** <0.001	*** <0.001	** 0.002	* 0.01	x	0.262	0.058	*** <0.001	-
	8N	43.400 ± 1.082	33.324 ± 1.467	37.466 ± 1.290	41.798 ± 1.506	25.845 ± 1.852	*** <0.001	*** <0.001	*** <0.001	*** <0.001	*** <0.001	*** <0.001	** 0.005	x	0.191
Ct.BS/BV [1/mm]	0N	9.836 ± 0.394	12.183 ± 0.351	11.627 ± 0.282	10.322 ± 0.194	-14.931 ± 1.937	*** <0.001	* 0.011	*** <0.001	*** <0.001	x	* 0.022	0.140	*** <0.001	-
	8N	9.677 ± 0.315	12.657 ± 0.632	11.352 ± 0.646	9.819 ± 0.679	-22.764 ± 1.593	*** <0.001	*** <0.001	*** <0.001	*** <0.001	*** <0.001	*** <0.001	*** <0.001	x	0.641
Ct.Th [μm]	0N	155.554 ± 5.396	137.670 ± 3.588	139.594 ± 3.126	148.587 ± 2.708	8.170 ± 1.617	*** <0.001	0.076	* 0.014	*** <0.001	x	0.261	1.000	*** <0.001	-

	8N	158.253 ± 4.356	136.541 ± 4.262	145.251 ± 4.391	159.885 ± 5.154	17.105 ± 1.448	*** <0.001	*** <0.001	*** <0.001	*** <0.001	*** <0.001	*** <0.001	*** <0.001	x	0.131
Full Strength [N]	0N	40.739 ± 1.673	29.305 ± 0.663	34.671 ± 1.123	41.160 ± 1.033	40.547 ± 2.331	*** <0.001	1.000	0.824	0.795	x	0.726	*** <0.001	*** <0.001	-
	8N	45.012 ± 1.838	29.215 ± 1.573	42.882 ± 1.414	52.445 ± 1.272	82.593 ± 8.998	*** <0.001	*** <0.001	*** <0.001	*** <0.001	*** <0.001	*** <0.001	* 0.027	x	**S* 0.026**
Full SED [kPa]	0N	0.908 ± 0.059	1.424 ± 0.055	1.164 ± 0.057	0.899 ± 0.030	-36.580 ± 1.702	*** <0.001	0.996	0.650	0.516	x	0.905	*** <0.001	*** <0.001	-
	8N	3.159 ± 0.190	5.730 ± 0.466	3.341 ± 0.134	2.467 ± 0.099	-55.681 ± 2.691	*** <0.001	*** <0.001	*** <0.001	*** <0.001	*** <0.001	*** <0.001	0.385	x	0.448
Full eff [με]	0N	306.378 ± 8.987	374.404 ± 6.860	344.760 ± 7.367	307.143 ± 4.588	-17.873 ± 1.068	*** <0.001	0.996	0.487	0.324	x	0.893	*** <0.001	*** <0.001	-
	8N	577.451 ± 16.650	738.907 ± 20.446	591.704 ± 11.876	514.655 ± 11.210	-30.134 ± 1.686	*** <0.001	*** <0.001	*** <0.001	*** <0.001	*** <0.001	*** <0.001	0.112	x	0.990

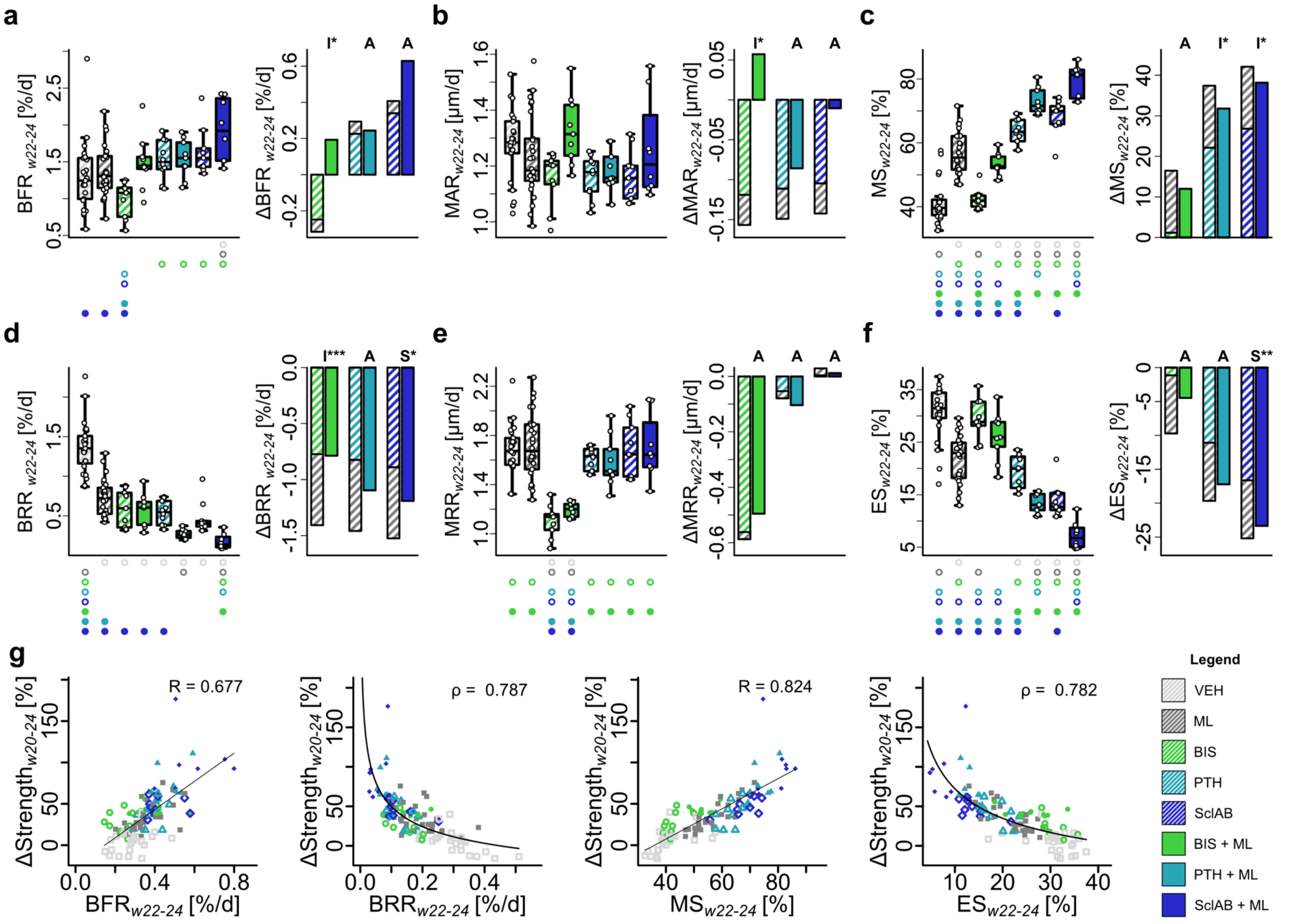
a
BFR w22-24 [%/d]
ΔBFR w22-24 [%/d]
I* A A
b
MARw22-24 [µm/d]
ΔMARw22-24 [µm/d]
I* A A
c
MSw22-24 [%]
ΔMSw22-24 [%]
A I* I*
d
BRR w22-24 [%/d]
ΔBRR w22-24 [%/d]
I*** A S*
e
MRRw22-24 [µm/d]
ΔMRRw22-24 [µm/d]
A A A
f
ESw22-24 [%]
ΔESw22-24 [%]
A A S**
g
ΔStrengthw20-24 [%]
R = 0.677
BFRw22-24 [%/d]
ρ = 0.787
BRRw22-24 [%/d]
R = 0.824
MSw22-24 [%]
ρ = 0.782
ESw22-24 [%]
Legend
VEH
ML
BIS
PTH
SclAB
BIS + ML
PTH + ML
SclAB + ML

Supplementary Figure S.2: Time interval w22-24: Mechanical loading increases bone formation and decreases resorption rates of anabolic and anti-catabolic pharmacological treatments which contribute to increased predicted strength. (a) bone formation rate per bone volume (BFR) **(b)** mineral apposition rate (MAR) **(c)** mineralizing surface per bone surface (MS) **(d)** bone resorption rate per bone volume (BRR) **(e)** mineral resorption rate (MRR) **(f)** eroded surface per bone surface (ES). **(a-f)** Each data point represents one animal (biological replicate; VEH: n=25, ML: n=26, BIS 0N: n=9, BIS 8N: n=9, PTH 0N: n=10, PTH 8N: n=9, SclAB 0N: n=9, SclAB 8N: n=8). The box spans the interquartile range (IQR) from the 25th to the 75th percentile. The median is shown as a line inside the box. Whiskers extend to the most extreme data points within 1.5 × IQR, points beyond this range are plotted as outliers. Δ denotes difference from VEH as no baseline value for dynamic bone morphometry rates was available. Bars show group means of sum of single vs. combined treatment indicating: antagonistic (I), additive (A) or synergistic (S) effects determined via the interaction term “treatment:loading” in a linear model on the $\log_2$-transformed values. Significances were calculated using one-way ANOVA followed by Tukey’s post-hoc test; significant differences are depicted with a symbol in the color of the corresponding group (o: to monotreatment, ●: to combined treatment, see Supplementary Table S.1 for color encoding, p<0.05). **(g)** Linear regression of ΔStrength with MS and BFR, power-law regression with ES and BRR in week interval w22-24. Source data are provided as a Source Data file.

Supplementary Dataset S.5: Dynamic morphometric parameters of the non-loaded (0N) and loaded (8N) trabecular compartments of 6th caudal vertebrae, given as group means ± standard error in week intervals w20-22 and w22-24. The %-change between week intervals w20-22 and w22-24, calculated per animal, is depicted as well. One-way ANOVA in each time interval for the shown animal groups followed by Tukey’s post-hoc test was used on the absolute values. ‘w20-22’ or ‘w22-24’ denotes the reference group; asterisks (*, **, ***) indicate groups that are significantly different from it. Significance is indicated as: *p<0.05, **p<0.01, ***p<0.001. Synergy (S) or antagonism (I) between treatment and mechanical loading was assessed using the interaction term “treatment:loading” from a fitted linear model applied to the $\log_2$-transformed absolute values within each interval.

Readout	N	w20-22	w22-24	%-change between w20-22 and w22-24 [%]	o	o	o	o	o	•	•	•	Synergy w20-22	Synergy w22-24
**VEH (0N: n=25; 8N: n=26)**														
BFR [%/d]	0N	1.081 ± 0.064	1.300 ± 0.092	29.102 ± 13.091	w20-22	** 0.008	0.767	* 0.035	0.467	* 0.022	*** <0.001	*** <0.001	-	-
					w22-24	0.998	0.345	0.715	0.256	0.872	0.675	** 0.001		
	8N	1.580 ± 0.079	1.367 ± 0.067	-9.603 ± 5.814	** 0.008	w20-22	0.956	1.000	0.998	0.996	** 0.001	*** <0.001	-	-
					0.998	w22-24	0.130	0.939	0.536	0.986	0.915	** 0.006		
BRR [%/d]	0N	2.044 ± 0.116	1.365 ± 0.065	-30.613 ± 3.390	w20-22	*** <0.001	*** <0.001	** 0.01	*** <0.001	*** <0.001	*** <0.001	*** <0.001	-	-
					w22-24	*** <0.001	*** <0.001	*** <0.001	*** <0.001	*** <0.001	*** <0.001	*** <0.001		
	8N	1.111 ± 0.056	0.730 ± 0.045	-32.952 ± 3.300	*** <0.001	w20-22	0.945	0.052	0.997	0.189	* 0.019	** 0.007	-	-
					*** <0.001	w22-24	0.790	0.374	0.100	0.700	*** <0.001	*** <0.001		
MAR [µm/d]	0N	1.307 ± 0.038	1.270 ± 0.026	-1.866 ± 2.070	w20-22	0.702	1.000	0.859	0.952	0.120	* 0.033	* 0.046	-	-
					w22-24	0.962	0.242	0.276	0.403	0.940	0.657	1.000		

	8N	1.378 ± 0.025	1.233 ± 0.029	-10.313 ± 1.841	0.702	w20-22	0.984	1.000	0.285	0.778	0.457	0.500	-	-
					0.962	w22-24	0.711	0.770	0.869	0.525	0.976	0.999		
MRR [µm/d]	0N	2.090 ± 0.048	1.691 ± 0.039	-18.593 ± 1.820	w20-22	** 0.01	*** <0.001	0.990	0.664	*** <0.001	*** <0.001	** 0.001	-	-
					w22-24	1.000	*** <0.001	0.971	1.000	*** <0.001	0.902	1.000		
	8N	1.905 ± 0.035	1.716 ± 0.052	-9.932 ± 2.175	** 0.01	w20-22	*** <0.001	0.554	0.986	*** <0.001	0.146	0.656	-	-
					1.000	w22-24	*** <0.001	0.877	1.000	*** <0.001	0.743	1.000		
MS [%]	0N	35.367 ± 0.953	41.220 ± 1.269	19.303 ± 6.190	w20-22	*** <0.001	* 0.02	*** <0.001	*** <0.001	*** <0.001	*** <0.001	*** <0.001	-	-
					w22-24	*** <0.001	0.999	*** <0.001	*** <0.001	*** <0.001	*** <0.001	*** <0.001		
	8N	50.426 ± 1.472	56.504 ± 1.328	12.979 ± 2.204	*** <0.001	w20-22	* 0.042	0.991	1.000	1.000	*** <0.001	*** <0.001	-	-
					*** <0.001	w22-24	*** <0.001	* 0.028	*** <0.001	0.792	*** <0.001	*** <0.001		
ES [%]	0N	33.408 ± 0.653	30.602 ± 0.974	-7.816 ± 3.031	w20-22	*** <0.001	0.958	0.569	*** <0.001	* 0.045	*** <0.001	*** <0.001	-	-
					w22-24	*** <0.001	0.996	*** <0.001	*** <0.001	0.105	*** <0.001	*** <0.001		
	8N	24.911 ± 1.149	22.035 ± 0.894	-10.109 ± 2.446	*** <0.001	w20-22	** 0.009	0.054	1.000	0.736	** 0.005	*** <0.001	-	-
					*** <0.001	w22-24	*** <0.001	0.717	*** <0.001	0.177	*** <0.001	*** <0.001		
**BIS (0N: n=9; 8N: n=9)**														
BFR [%/d]	0N	1.375 ± 0.063	0.984 ± 0.083	-27.677 ± 6.537	0.767	0.956	w20-22	0.897	1.000	0.807	*** <0.001	*** <0.001	-	-
					0.345	0.130	w22-24	* 0.034	** 0.006	0.072	* 0.032	*** <0.001		
	8N	1.716 ± 0.156	1.493 ± 0.124	-10.502 ± 6.308	* 0.022	0.996	0.807	1.000	0.952	w20-22	0.085	* 0.033	0.183	**I* 0.018**
					0.872	0.986	0.072	1.000	0.990	w22-24	1.000	0.227		
BRR [%/d]	0N	0.947 ± 0.120	0.592 ± 0.079	-36.308 ± 5.376	*** <0.001	0.945	w20-22	* 0.018	0.800	0.943	0.564	0.346	-	-
					*** <0.001	0.790	w22-24	1.000	0.962	1.000	0.077	** 0.009		
	8N	0.745 ± 0.108	0.578 ± 0.068	-18.239 ± 7.643	*** <0.001	0.189	0.943	*** <0.001	0.147	w20-22	0.996	0.954	**I* 0.017**	**I*** <0.001**
					*** <0.001	0.700	1.000	1.000	0.981	w22-24	0.104	* 0.013		
MAR [µm/d]	0N	1.325 ± 0.025	1.151 ± 0.033	-12.895 ± 2.740	1.000	0.984	w20-22	0.987	0.940	0.472	0.239	0.264	-	-
					0.242	0.711	w22-24	1.000	1.000	0.072	0.999	0.643		
	8N	1.469 ± 0.048	1.328 ± 0.041	-9.312 ± 2.371	0.120	0.778	0.472	0.932	* 0.039	w20-22	1.000	1.000	0.531	**I** 0.002**
					0.940	0.525	0.072	0.083	0.130	w22-24	0.253	0.955		
MRR [µm/d]	0N	1.262 ± 0.045	1.104 ± 0.045	-12.340 ± 2.781	*** <0.001	*** <0.001	w20-22	*** <0.001	*** <0.001	1.000	*** <0.001	*** <0.001	-	-
					*** <0.001	*** <0.001	w22-24	*** <0.001	*** <0.001	0.981	*** <0.001	*** <0.001		
	8N	1.234 ± 0.041	1.195 ± 0.019	-2.511 ± 2.853	*** <0.001	*** <0.001	1.000	*** <0.001	*** <0.001	w20-22	*** <0.001	*** <0.001	0.191	0.176
					*** <0.001	*** <0.001	0.981	*** <0.001	*** <0.001	w22-24	** 0.003	*** <0.001		
MS [%]	0N	43.210 ± 1.460	42.389 ± 1.092	-1.195 ± 3.476	* 0.02	* 0.042	w20-22	0.494	0.128	0.373	*** <0.001	*** <0.001	-	-

					0.999	*** <0.001	w22-24	*** <0.001	*** <0.001	** 0.002	*** <0.001	*** <0.001		
	8N	49.278 ± 1.266	53.222 ± 1.160	8.439 ± 3.108	*** <0.001	1.000	0.373	1.000	0.999	w20-22	*** <0.001	*** <0.001	**I** 0.002**	0.121
					*** <0.001	0.792	** 0.002	** 0.004	*** <0.001	w22-24	*** <0.001	*** <0.001		
ES [%]	0N	31.464 ± 1.830	29.459 ± 1.287	-5.278 ± 3.625	0.958	** 0.009	w20-22	0.999	* 0.024	0.694	*** <0.001	*** <0.001	-	-
					0.996	*** <0.001	w22-24	*** <0.001	*** <0.001	0.671	*** <0.001	*** <0.001		
	8N	27.798 ± 1.385	26.123 ± 1.562	-5.820 ± 4.220	* 0.045	0.736	0.694	0.950	0.711	w20-22	*** <0.001	*** <0.001	0.075	0.067
					0.105	0.177	0.671	* 0.015	*** <0.001	w22-24	*** <0.001	*** <0.001		
**PTH (0N: n=10; 8N: n=9)**														
BFR [%/d]	0N	1.664 ± 0.140	1.526 ± 0.084	-4.375 ± 7.003	* 0.035	1.000	0.897	w20-22	0.984	1.000	* 0.037	* 0.013	-	-
					0.715	0.939	* 0.034	w22-24	0.997	1.000	1.000	0.291		
	8N	2.374 ± 0.235	1.543 ± 0.088	-30.211 ± 7.468	*** <0.001	** 0.001	*** <0.001	* 0.037	** 0.003	0.085	w20-22	1.000	0.741	0.635
					0.675	0.915	* 0.032	1.000	0.999	1.000	w22-24	0.379		
BRR [%/d]	0N	1.537 ± 0.078	0.541 ± 0.048	-64.449 ± 3.282	** 0.01	0.052	* 0.018	w20-22	0.547	*** <0.001	*** <0.001	*** <0.001	-	-
					*** <0.001	0.374	1.000	w22-24	0.999	1.000	0.194	* 0.028		
	8N	0.617 ± 0.049	0.268 ± 0.020	-55.085 ± 4.167	*** <0.001	* 0.019	0.564	*** <0.001	* 0.021	0.996	w20-22	1.000	**S* 0.026**	0.814
					*** <0.001	*** <0.001	0.077	0.194	0.576	0.104	w22-24	0.991		
MAR [µm/d]	0N	1.386 ± 0.035	1.159 ± 0.024	-16.012 ± 2.310	0.859	1.000	0.987	w20-22	0.437	0.932	0.743	0.761	-	-
					0.276	0.770	1.000	w22-24	1.000	0.083	1.000	0.698		
	8N	1.498 ± 0.041	1.185 ± 0.025	-20.591 ± 2.008	* 0.033	0.457	0.239	0.743	* 0.012	1.000	w20-22	1.000	0.836	0.361
					0.657	0.976	0.999	1.000	1.000	0.253	w22-24	0.922		
MRR [µm/d]	0N	2.033 ± 0.037	1.612 ± 0.029	-20.505 ± 1.824	0.990	0.554	*** <0.001	w20-22	0.993	*** <0.001	** 0.006	0.068	-	-
					0.971	0.877	*** <0.001	w22-24	0.989	*** <0.001	1.000	0.984		
	8N	1.719 ± 0.073	1.587 ± 0.069	-6.995 ± 3.963	*** <0.001	0.146	*** <0.001	** 0.006	0.08	*** <0.001	w20-22	0.998	0.128	0.727
					0.902	0.743	*** <0.001	1.000	0.957	** 0.003	w22-24	0.945		
MS [%]	0N	48.606 ± 1.410	63.343 ± 1.250	31.128 ± 3.855	*** <0.001	0.991	0.494	w20-22	0.992	1.000	*** <0.001	*** <0.001	-	-
					*** <0.001	* 0.028	*** <0.001	w22-24	0.601	** 0.004	** 0.006	*** <0.001		
	8N	64.556 ± 2.224	73.002 ± 1.396	13.822 ± 3.229	*** <0.001	*** <0.001	*** <0.001	*** <0.001	*** <0.001	*** <0.001	w20-22	1.000	0.334	**I** 0.002**
					*** <0.001	*** <0.001	*** <0.001	** 0.006	0.557	*** <0.001	w22-24	0.274		
ES [%]	0N	30.173 ± 1.288	19.512 ± 0.969	-34.805 ± 3.436	0.569	0.054	0.999	w20-22	0.100	0.950	*** <0.001	*** <0.001	-	-
					*** <0.001	0.717	*** <0.001	w22-24	0.077	* 0.015	* 0.033	*** <0.001		
	8N	18.071 ± 1.458	13.395 ± 0.637	-22.858 ± 5.636	*** <0.001	** 0.005	*** <0.001	*** <0.001	0.102	*** <0.001	w20-22	0.383	**S* 0.028**	0.620
					*** <0.001	*** <0.001	*** <0.001	* 0.033	1.000	*** <0.001	w22-24	0.053		

**SclAB (0N: n=9; 8N: n=8)**														
BFR [%/d]	0N	1.462 ± 0.136	1.639 ± 0.109	20.318 ± 15.922	0.467	0.998	1.000	0.984	w20-22	0.952	** 0.003	*** <0.001	-	-
					0.256	0.536	** 0.006	0.997	w22-24	0.990	0.999	0.731		
	8N	2.477 ± 0.351	1.928 ± 0.150	-16.789 ± 6.327	*** <0.001	*** <0.001	*** <0.001	* 0.013	*** <0.001	* 0.033	1.000	w20-22	0.525	0.594
					** 0.001	** 0.006	*** <0.001	0.291	0.731	0.227	0.379	w22-24		
BRR [%/d]	0N	1.211 ± 0.081	0.474 ± 0.069	-60.942 ± 4.569	*** <0.001	0.997	0.800	0.547	w20-22	0.147	* 0.021	** 0.009	-	-
					*** <0.001	0.100	0.962	0.999	w22-24	0.981	0.576	0.155		
	8N	0.545 ± 0.061	0.174 ± 0.034	-67.050 ± 6.237	*** <0.001	** 0.007	0.346	*** <0.001	** 0.009	0.954	1.000	w20-22	0.069	**S* 0.028**
					*** <0.001	*** <0.001	** 0.009	* 0.028	0.155	* 0.013	0.991	w22-24		
MAR [µm/d]	0N	1.242 ± 0.024	1.166 ± 0.031	-5.981 ± 2.605	0.952	0.285	0.940	0.437	w20-22	* 0.039	* 0.012	* 0.015	-	-
					0.403	0.869	1.000	1.000	w22-24	0.130	1.000	0.787		
	8N	1.499 ± 0.090	1.260 ± 0.063	-15.104 ± 3.318	* 0.046	0.500	0.264	0.761	* 0.015	1.000	1.000	w20-22	**S* 0.047**	0.070
					1.000	0.999	0.643	0.698	0.787	0.955	0.922	w22-24		
MRR [µm/d]	0N	1.968 ± 0.031	1.694 ± 0.075	-13.971 ± 3.383	0.664	0.986	*** <0.001	0.993	w20-22	*** <0.001	0.080	0.382	-	-
					1.000	1.000	*** <0.001	0.989	w22-24	*** <0.001	0.957	1.000		
	8N	1.778 ± 0.049	1.703 ± 0.093	-4.080 ± 5.144	** 0.001	0.656	*** <0.001	0.068	0.382	*** <0.001	0.998	w20-22	0.845	0.970
					1.000	1.000	*** <0.001	0.984	1.000	*** <0.001	0.945	w22-24		
MS [%]	0N	50.789 ± 2.387	68.026 ± 1.861	35.700 ± 5.946	*** <0.001	1.000	0.128	0.992	w20-22	0.999	*** <0.001	*** <0.001	-	-
					*** <0.001	*** <0.001	*** <0.001	0.601	w22-24	*** <0.001	0.557	** 0.001		
	8N	65.647 ± 1.951	79.382 ± 1.799	21.229 ± 2.148	*** <0.001	*** <0.001	*** <0.001	*** <0.001	*** <0.001	*** <0.001	1.000	w20-22	0.222	**I** 0.005**
					*** <0.001	*** <0.001	*** <0.001	*** <0.001	** 0.001	*** <0.001	0.274	w22-24		
ES [%]	0N	24.190 ± 1.589	13.979 ± 1.099	-41.486 ± 3.476	*** <0.001	1.000	* 0.024	0.100	w20-22	0.711	0.102	*** <0.001	-	-
					*** <0.001	*** <0.001	*** <0.001	0.077	w22-24	*** <0.001	1.000	* 0.023		
	8N	13.231 ± 1.197	7.259 ± 0.929	-43.256 ± 8.665	*** <0.001	*** <0.001	*** <0.001	*** <0.001	*** <0.001	*** <0.001	0.383	w20-22	**S** 0.005**	**S*** <0.001**
					*** <0.001	*** <0.001	*** <0.001	*** <0.001	* 0.023	*** <0.001	0.053	w22-24		

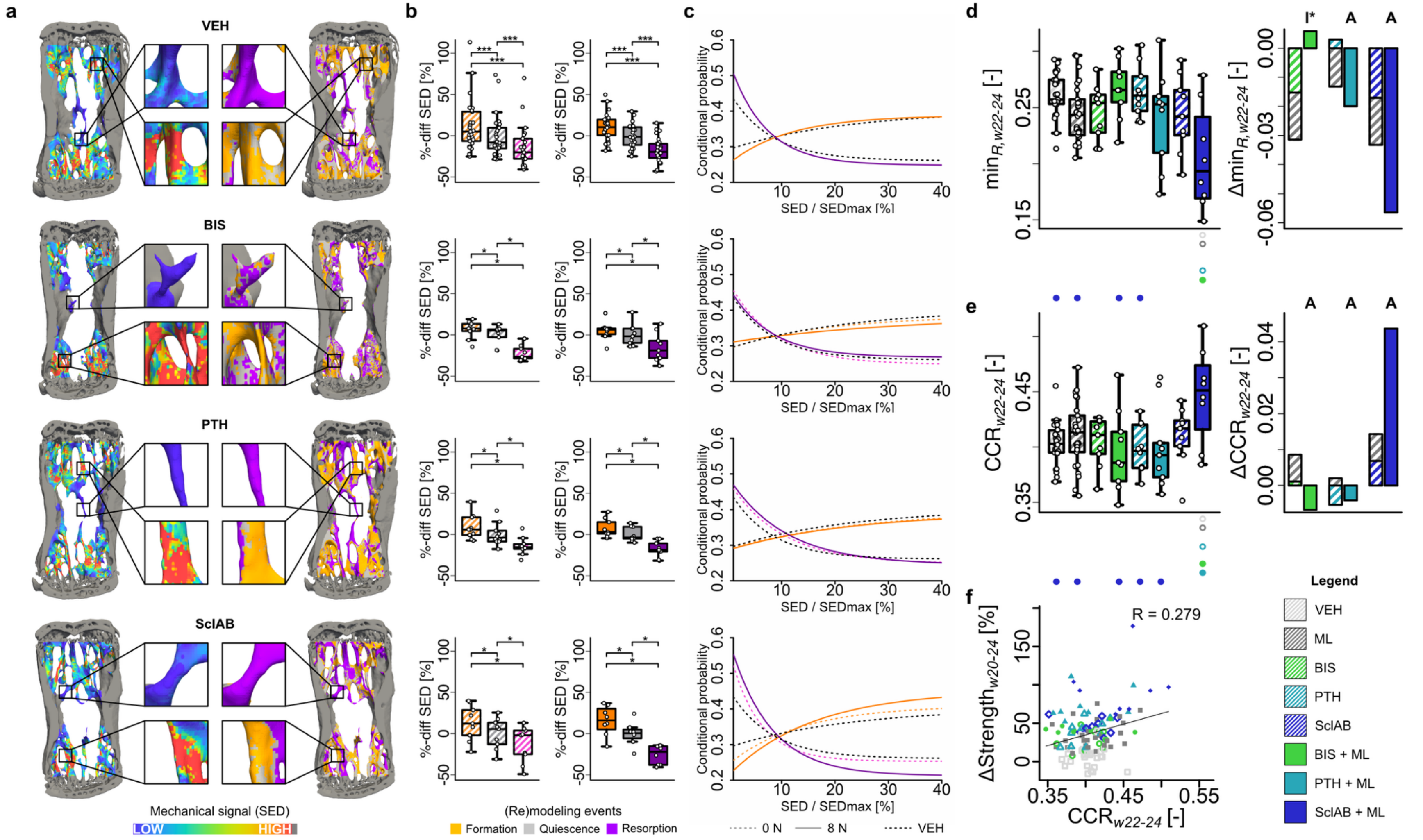

Supplementary Figure S.3: Formation aligns with high mechanical signal, resorption with low: (a) Spatial comparison of local mechanical signal (SED at week 20) with sites of trabecular bone formation and resorption (w22-24). **(b)** SED at formation, resorption, and quiescent sites[2], normalized to the group mean of quiescence to allow statistical testing with this group. Each data point represents one animal (biological replicate; VEH: n=25, ML: n=26, BIS 0N: n=9, BIS 8N: n=9, PTH 0N: n=10, PTH 8N: n=9, SclAB 0N: n=9, SclAB 8N: n=8). The box spans the interquartile range (IQR) from the 25th to the 75th percentile. The median is shown as a line inside the box. Whiskers extend to the most extreme data points

within 1.5 × IQR, points beyond this range are plotted as outliers. *p<0.05, ***<0.001 using two-sided paired Wilcoxon-signed rank test with Bonferroni correction; exact p-values are found in Supplementary Table S.6. **(c)** The plots show the exponential fitting functions (taking all animals per group into account) for conditional probability of formation and resorption as a function of SED/SEDmax. Dashed lines represent monotreatment (0N, including VEH), and solid lines represent combined treatment with mechanical loading (8N, including ML). VEH is indicated by black dotted line. Values are cropped above 40% due to low voxel count[2]. **(d-e)** Each data point represents one animal (biological replicate; VEH: n=25, ML: n=26, BIS 0N: n=9, BIS 8N: n=9, PTH 0N: n=10, PTH 8N: n=9, SclAB 0N: n=9, SclAB 8N: n=8). The box spans the interquartile range (IQR) from the 25th to the 75th percentile. The median is shown as a line inside the box. Whiskers extend to the most extreme data points within 1.5 × IQR, points beyond this range are plotted as outliers. [-] denotes unitless, Δ denotes change from VEH; sum of single vs. combined treatment group means indicating antagonistic (I), additive (A) or synergistic (S) effects. **(d)** Asymptotic resorption probability value) **(e)** CCR as a measure of mechanoregulated (re)modeling **(f)** Linear regression analysis in week interval w22-24 of ΔStrength with CCR. Significant differences are depicted with a symbol in the color of the corresponding group (o: to monotreatment, ●: to combined treatment, see Supplementary Table S.1 for color encoding, p<0.05). Source data are provided as a Source Data file.

Supplementary Dataset S.6: Group means ± standard error of the absolute strain energy density (SED) values in formation (F), quiescence (Q) and resorption (R) sites are reported for all vehicle and treatment groups for both time intervals. Furthermore, mean SED at formation, quiescent and resorption sites expressed as percent difference from the mean (over all animals) quiescent SED are reported as |F-Q| and |Q-R|. Normalization to the mean (over all animals) quiescent SED preserves group properties thus allows statistical comparison of relative differences between F,Q,R regions. P-values were calculated using pairwise two-sided Wilcoxon signed-rank tests with Bonferroni correction. Significance is indicated as: *p<0.05, **p<0.01, ***p<0.001.

Group	Week	F [kPa]	Q [kPa]	R [kPa]	|F-Q| [%]	|Q-R| [%]	p-value (F vs. Q)	p-value (Q vs. R)
VEH 0N	20-22	3.219 ± 0.270	2.882 ± 0.216	2.453 ± 0.157	-9.466 ± 1.079	-13.912 ± 1.149	*** **<0.001**	*** **<0.001**
	22-24	3.182 ± 0.178	2.820 ± 0.146	2.430 ± 0.140	-10.818 ± 1.105	-14.064 ± 1.245	*** **<0.001**	*** **<0.001**
VEH 8N	20-22	11.007 ± 0.474	9.575 ± 0.362	7.649 ± 0.349	-12.358 ± 1.499	-20.396 ± 1.563	*** **<0.001**	*** **<0.001**
	22-24	8.184 ± 0.240	7.433 ± 0.211	6.107 ± 0.224	-9.008 ± 0.944	-17.932 ± 1.761	*** **<0.001**	*** **<0.001**
BIS 0N	20-22	2.962 ± 0.203	2.765 ± 0.176	2.269 ± 0.143	-6.275 ± 1.429	-17.742 ± 2.013	* **0.012**	* **0.012**
	22-24	2.282 ± 0.077	2.143 ± 0.082	1.663 ± 0.066	-6.176 ± 1.235	-21.962 ± 2.789	* **0.023**	* **0.012**
BIS 8N	20-22	10.552 ± 0.469	9.836 ± 0.433	8.268 ± 0.552	-6.640 ± 1.729	-16.333 ± 3.029	* **0.023**	* **0.012**
	22-24	7.409 ± 0.265	7.066 ± 0.311	5.998 ± 0.407	-4.542 ± 2.560	-15.696 ± 2.612	n.s. 0.750	* **0.012**
PTH 0N	20-22	3.142 ± 0.257	2.841 ± 0.210	2.291 ± 0.186	-9.022 ± 1.643	-19.408 ± 1.848	* **0.012**	** **0.006**
	22-24	2.353 ± 0.100	2.162 ± 0.092	1.854 ± 0.071	-8.051 ± 1.232	-14.092 ± 1.161	** **0.006**	** **0.006**

PTH 8N	20-22	10.192 ± 0.672	8.341 ± 0.481	6.661 ± 0.501	-17.642 ± 2.301	-20.566 ± 1.610	* **0.012**	* **0.012**
	22-24	5.835 ± 0.199	5.435 ± 0.157	4.469 ± 0.175	-6.673 ± 1.225	-17.568 ± 3.207	* **0.012**	* **0.023**
SclAB 0N	20-22	3.185 ± 0.213	2.654 ± 0.172	2.246 ± 0.145	-16.350 ± 2.545	-15.293 ± 1.466	* **0.012**	* **0.012**
	22-24	2.333 ± 0.148	2.069 ± 0.128	1.799 ± 0.152	-11.208 ± 0.866	-13.966 ± 3.199	* **0.012**	* **0.023**
SclAB 8N	20-22	13.647 ± 1.477	11.073 ± 1.173	8.818 ± 1.399	-18.578 ± 1.754	-22.010 ± 4.365	* **0.023**	* **0.023**
	22-24	6.971 ± 0.380	6.032 ± 0.296	4.487 ± 0.248	-12.978 ± 2.650	-25.266 ± 3.306	* **0.023**	* **0.023**

Supplementary Dataset S.7: Saturating exponential curve fits for formation slope values were conducted by fitting the function $f(x) = y_0 + a \cdot (1 - \exp(-b \cdot x))$ to the first 40% of the normalized SED/SED_{max} interval for each animal individually. Coefficients are presented as group means ± standard error. Parameter y_0 reflects the level of formation in the absence of mechanical stimulus (i.e. at SED = 0), representing non-targeted formation. Coefficient a indicates the maximal increase in formation in response to SED, while $y_0 + a$ defines the plateau, or the maximum potential formation probability at high SED. b represents the sensitivity or rate of response, i.e. higher b-values indicate that the curve rises more quickly, reaching the plateau earlier (i.e. formation increases rapidly with even low SED). Synergy (S) or antagonism (I) between treatment and mechanical loading was assessed using the interaction term "treatment:loading" from a fitted linear model applied to the log_2-transformed absolute values within each interval. P-values were obtained using one-way ANOVA in each time interval for the shown groups followed by Tukey's post-hoc test to account for multiple comparisons. 'w20-22' or 'w22-24' denotes the reference group; asterisks (*, **, ***) indicate groups that are significantly different from it. Significance is indicated as: *p<0.05, **p<0.01, ***p<0.001.

Readout	N	w20-22	w22-24	%-change between w20-22 and w22-24 [%]	○	○	○	○	○	●	●	●	Synergy w20-22	Synergy w22-24
**VEH (0N: n=25; 8N: n=26)**														
y0 (level of formation at SED=0)	0N	0.297 ± 0.004	0.291 ± 0.005	-1.974 ± 1.475	w20-22	* **0.020**	0.997	0.721	* **0.014**	0.999	*** **<0.001**	*** **<0.001**	-	-
					w22-24	** **0.003**	1.000	0.920	** **0.002**	0.991	* **0.029**	*** **<0.001**		
	8N	0.269 ± 0.007	0.251 ± 0.009	-6.011 ± 2.847	* **0.020**	w20-22	* **0.042**	0.984	0.959	0.056	0.518	0.157	-	-
					** **0.003**	w22-24	0.051	0.637	0.907	** **0.007**	1.000	0.181		
a (maximum increase in formation)	0N	0.170 ± 0.036	0.182 ± 0.031	-9.563 ± 73.721	w20-22	0.774	0.998	1.000	0.996	0.838	** **0.005**	0.965	-	-
					w22-24	1.000	1.000	1.000	0.977	1.000	0.767	0.302		
	8N	0.251 ± 0.038	0.166 ± 0.022	4.991 ± 17.610	0.774	w20-22	1.000	0.748	1.000	0.179	0.119	1.000	-	-
					1.000	w22-24	1.000	1.000	0.921	1.000	0.605	0.187		
b (sensitivity of response)	0N	0.035 ± 0.006	0.044 ± 0.007	182.258 ± 85.085	w20-22	0.889	1.000	0.956	0.971	1.000	0.992	0.276	-	-
					w22-24	* **0.028**	1.000	1.000	0.172	0.999	0.887	0.997		
	8N	0.048 ± 0.008	0.084 ± 0.009	378.359 ± 131.954	0.889	w20-22	0.955	1.000	1.000	0.898	1.000	0.831	-	-

					* 0.028	w22-24	0.221	0.299	1.000	0.076	0.963	0.760		
y0+a (plateau at high mechanical strains)	0N	0.467 ± 0.035	0.473 ± 0.031	8.536 ± 7.584	w20-22	0.970	0.994	0.997	1.000	0.874	* 0.039	1.000	-	-
					w22-24	0.942	1.000	1.000	1.000	1.000	0.984	0.918		
	8N	0.520 ± 0.038	0.417 ± 0.020	-11.339 ± 6.734	0.970	w20-22	1.000	0.821	0.999	0.421	0.222	1.000	-	-
					0.942	w22-24	0.985	0.998	0.976	0.999	0.652	0.457		
**BIS (0N: n=9; 8N: n=9)**														
y0	0N	0.305 ± 0.003	0.293 ± 0.007	-3.918 ± 1.831	0.997	* 0.042	w20-22	0.540	* 0.016	1.000	** 0.001	*** <0.001	-	-
					1.000	0.051	w22-24	0.940	* 0.012	0.999	0.090	*** <0.001		
	8N	0.304 ± 0.010	0.302 ± 0.011	-0.043 ± 4.188	0.999	0.056	1.000	0.601	* 0.021	w20-22	** 0.002	*** <0.001	0.124	**I* 0.020**
					0.991	** 0.007	0.999	0.682	** 0.002	w22-24	* 0.021	*** <0.001		
a	0N	0.218 ± 0.065	0.184 ± 0.061	77.465 ± 69.861	0.998	1.000	w20-22	0.984	1.000	0.665	0.156	1.000	-	-
					1.000	1.000	w22-24	1.000	0.994	1.000	0.908	0.553		
	8N	0.066 ± 0.056	0.151 ± 0.047	-326.699 ± 212.075	0.838	0.179	0.665	0.988	0.640	w20-22	*** <0.001	0.468	0.206	0.683
					1.000	1.000	1.000	1.000	0.932	w22-24	0.699	0.303		
b	0N	0.033 ± 0.009	0.042 ± 0.014	658.509 ± 610.023	1.000	0.955	w20-22	0.971	0.980	1.000	0.993	0.433	-	-
					1.000	0.221	w22-24	1.000	0.362	1.000	0.942	0.998		
	8N	0.030 ± 0.011	0.035 ± 0.013	164.000 ± 138.631	1.000	0.898	1.000	0.936	0.952	w20-22	0.980	0.339	0.444	**S* 0.015**
					0.999	0.076	1.000	0.999	0.175	w22-24	0.785	0.970		
y0+a	0N	0.523 ± 0.067	0.478 ± 0.061	5.029 ± 19.776	0.994	1.000	w20-22	0.920	0.999	0.644	0.498	1.000	-	-
					1.000	0.985	w22-24	1.000	1.000	1.000	0.997	0.976		
	8N	0.370 ± 0.049	0.453 ± 0.047	145.241 ± 137.128	0.874	0.421	0.644	0.999	0.918	w20-22	** 0.007	0.897	**I** 0.007**	0.682
					1.000	0.999	1.000	1.000	1.000	w22-24	0.973	0.905		
**PTH (0N: n=10; 8N: n=9)**														
y0	0N	0.279 ± 0.007	0.275 ± 0.008	-1.031 ± 3.629	0.721	0.984	0.540	w20-22	0.716	0.601	0.260	0.701	-	-
					0.920	0.637	0.940	w22-24	0.204	0.682	0.636	* 0.012		
	8N	0.246 ± 0.009	0.246 ± 0.010	0.629 ± 4.501	*** 0.001	0.518	** 0.001	0.260	0.996	** 0.002	w20-22	0.998	0.600	0.631
					* 0.029	1.000	0.090	0.636	0.996	* 0.021	w22-24	0.612		
a	0N	0.140 ± 0.022	0.185 ± 0.043	41.451 ± 27.850	1.000	0.748	0.984	w20-22	0.980	0.988	* 0.011	0.918	-	-
					1.000	1.000	1.000	w22-24	0.993	1.000	0.897	0.524		
	8N	0.449 ± 0.111	0.288 ± 0.101	-2.790 ± 38.514	** 0.005	0.119	0.156	* 0.011	0.169	*** <0.001	w20-22	0.354	0.119	0.360
					0.767	0.605	0.908	0.897	1.000	0.699	w22-24	0.998		
b	0N	0.050 ± 0.008	0.046 ± 0.010	-3.976 ± 23.171	0.956	1.000	0.971	w20-22	1.000	0.936	1.000	0.944	-	-

					1.000	0.299	1.000	w22-24	0.455	0.999	0.977	1.000		
	8N	0.046 ± 0.016	0.066 ± 0.013	456.616 ± 263.598	0.992	1.000	0.993	1.000	1.000	0.980	w20-22	0.891	0.152	0.399
					0.887	0.963	0.942	0.977	0.966	0.785	w22-24	1.000		
y0+a	0N	0.419 ± 0.020	0.459 ± 0.043	8.605 ± 6.560	0.997	0.821	0.920	w20-22	0.997	0.999	* 0.031	0.994	-	-
					1.000	0.998	1.000	w22-24	1.000	1.000	0.981	0.921		
	8N	0.695 ± 0.115	0.534 ± 0.100	-9.314 ± 19.146	* 0.039	0.222	0.498	* 0.031	0.204	** 0.007	w20-22	0.285	0.101	0.220
					0.984	0.652	0.997	0.981	0.998	0.973	w22-24	1.000		
**SclAB (0N: n=9; 8N: n=8)**														
y0	0N	0.256 ± 0.012	0.234 ± 0.015	-8.253 ± 4.550	* 0.014	0.959	* 0.016	0.716	w20-22	* 0.021	0.996	0.886	-	-
					** 0.002	0.907	* 0.012	0.204	w22-24	** 0.002	0.996	0.950		
	8N	0.237 ± 0.016	0.215 ± 0.018	-6.395 ± 10.024	*** <0.001	0.157	*** <0.001	0.071	0.886	*** <0.001	0.998	w20-22	0.819	0.450
					*** <0.001	0.181	*** <0.001	* 0.012	0.950	*** <0.001	0.6122	w22-24		
a	0N	0.221 ± 0.036	0.248 ± 0.067	22.782 ± 32.467	0.996	1.000	1.000	0.980	w20-22	0.640	0.169	1.000	-	-
					0.977	0.921	0.994	0.993	w22-24	0.932	1.000	0.945		
	8N	0.249 ± 0.038	0.345 ± 0.081	54.781 ± 35.118	0.965	1.000	1.000	0.918	1.000	0.468	0.354	w20-22	0.470	0.258
					0.302	0.187	0.553	0.524	0.945	0.303	0.998	w22-24		
b	0N	0.049 ± 0.012	0.087 ± 0.022	102.185 ± 51.133	0.971	1.000	0.980	1.000	w20-22	0.952	1.000	0.942	-	-
					0.172	1.000	0.362	0.455	w22-24	0.175	0.966	0.814		
	8N	0.069 ± 0.018	0.056 ± 0.013	47.010 ± 58.956	0.276	0.831	0.433	0.944	0.942	0.339	0.891	w20-22	0.882	**I* 0.032**
					0.997	0.760	0.998	1.000	0.814	0.970	1.000	w22-24		
y0+a	0N	0.478 ± 0.033	0.483 ± 0.070	2.365 ± 13.366	1.000	0.999	0.999	0.997	w20-22	0.918	0.204	1.000	-	-
					1.000	0.976	1.000	1.000	w22-24	1.000	0.998	0.983		
	8N	0.486 ± 0.034	0.560 ± 0.083	21.002 ± 22.630	1.000	1.000	1.000	0.994	1.000	0.897	0.285	w20-22	0.676	0.107
					0.918	0.457	0.976	0.921	0.983	0.905	1.000	w22-24		

Supplementary Dataset S.8: Decaying exponential curve fits for resorption were conducted by fitting the function $f(x) = y_0 + a \cdot \exp(-b \cdot x)$ to the first 40% of the normalized SED/SED_{max} interval for each animal individually. The fitted coefficients for each animal are presented as group means ± standard error. Resorption asymptote min_R represents the saturation level of resorption at high mechanical signal, i.e. the minimum resorption probability ($x \to \infty$), indicative of the amount of non-targeted resorption. Coefficient a represents the magnitude of the response, i.e. the difference between the initial and the minimum resorption probability. $a + y_0$ represents the peak resorption probability at low SED. Coefficient b is the rate of decay, representing how quickly the probability of resorption drops as SED increases. Synergy (S) or antagonism (I) between treatment and mechanical loading was assessed using the interaction term “treatment:loading” from a fitted linear model applied to the log_2-transformed absolute values within each interval. P-values were obtained using one-way ANOVA in each time interval for the shown groups followed by Tukey’s post-hoc test to account for multiple comparisons. ‘w20-22’ or ‘w22-24’ denotes the reference group; asterisks (*, **, ***) indicate groups that are significantly different from it. Significance is indicated as: *p<0.05, **p<0.01, ***p<0.001.

Readout	N	w20-22	w22-24	%-change between w20-22 and w22-24 [%]	○	○	○	○	○	●	●	●	Synergy w20-22	Synergy w22-24
**VEH (0N: n=25; 8N: n=26)**														
min_R (saturation level of resorption; plateau at high mechanical strains)	0N	0.266 ± 0.003	0.261 ± 0.004	-1.845 ± 1.304	w20-22	* 0.020	0.977	0.997	0.740	0.549	** 0.002	0.070	-	-
					w22-24	0.474	0.867	1.000	0.786	0.999	0.625	*** <0.001		
	8N	0.233 ± 0.006	0.245 ± 0.005	7.366 ± 5.030	* 0.020	w20-22	0.804	0.579	0.989	0.999	0.738	0.999	-	-
					0.474	w22-24	1.000	0.622	1.000	0.490	1.000	* 0.016		
a (magnitude of response)	0N	0.200 ± 0.010	0.208 ± 0.011	6.873 ± 6.119	w20-22	** 0.002	0.999	0.596	0.067	1.000	** 0.006	*** <0.001	-	-
					w22-24	*** <0.001	0.968	0.988	* 0.012	1.000	* 0.029	*** <0.001		
	8N	0.274 ± 0.014	0.304 ± 0.015	15.330 ± 6.368	** 0.002	w20-22	* 0.015	0.928	1.000	* 0.037	0.988	0.159	-	-
					*** <0.001	w22-24	0.204	0.105	1.000	* 0.018	1.000	* 0.013		
b (sensitivity of decay)	0N	0.144 ± 0.006	0.145 ± 0.008	6.571 ± 7.975	w20-22	0.910	0.712	1.000	1.000	0.407	0.332	0.491	-	-
					w22-24	0.988	0.917	0.884	0.997	1.000	0.667	1.000		
	8N	0.130 ± 0.008	0.157 ± 0.012	832.154 ± 820.041	0.910	w20-22	0.995	1.000	0.978	0.924	0.878	0.088	-	-
					0.988	w22-24	0.571	0.491	1.000	1.000	0.269	0.999		
y0+a (peak resorption under zero mechanical loading)	0N	0.467 ± 0.008	0.469 ± 0.009	0.897 ± 2.038	w20-22	0.126	0.896	0.639	0.174	0.783	0.495	*** <0.001	-	-
					w22-24	*** <0.001	0.999	0.898	** 0.005	1.000	* 0.024	*** <0.001		
	8N	0.507 ± 0.013	0.548 ± 0.012	9.476 ± 3.470	0.126	w20-22	* 0.030	1.000	0.998	* 0.014	1.000	0.110	-	-
					*** <0.001	w22-24	* 0.033	0.130	1.000	* 0.018	1.000	0.107		
**BIS (0N: n=9; 8N: n=9)**														
min_R	0N	0.253 ± 0.009	0.246 ± 0.008	-2.098 ± 4.855	0.977	0.804	w20-22	1.000	0.999	0.994	0.183	0.682	-	-

					0.867	1.000	w22-24	0.860	1.000	0.766	1.000	0.071		
	8N	0.241 ± 0.022	0.267 ± 0.009	26.748 ± 24.035	0.549	0.999	0.994	0.968	1.000	w20-22	0.629	0.979	0.692	**I* 0.027**
					0.999	0.490	0.766	1.000	0.684	w22-24	0.542	*** **<0.001**		
a	0N	0.186 ± 0.016	0.236 ± 0.018	30.103 ± 9.802	0.999	* **0.015**	w20-22	0.505	0.078	1.000	* **0.012**	*** **<0.001**	-	-
					0.968	0.204	w22-24	1.000	0.421	0.994	0.577	*** **<0.001**		
	8N	0.194 ± 0.031	0.211 ± 0.022	27.676 ± 17.435	1.000	* **0.037**	1.000	0.673	0.141	w20-22	* **0.025**	*** **<0.001**	**I* 0.024**	**I** 0.004**
					1.000	* **0.018**	0.994	0.998	0.088	w22-24	0.154	*** **<0.001**		
b	0N	0.119 ± 0.014	0.121 ± 0.008	22.051 ± 22.906	0.712	0.995	w20-22	0.965	0.848	1.000	1.000	0.065	-	-
					0.917	0.571	w22-24	1.000	0.761	0.864	1.000	0.975		
	8N	0.112 ± 0.016	0.153 ± 0.022	156.850 ± 136.350	0.407	0.924	1.000	0.834	0.624	w20-22	1.000	* **0.023**	0.807	0.589
					1.000	1.000	0.864	0.830	1.000	w22-24	0.632	1.000		
y0+a	0N	0.440 ± 0.013	0.482 ± 0.010	10.034 ± 2.672	0.896	* **0.030**	w20-22	0.189	* **0.037**	1.000	0.131	*** **<0.001**	-	-
						*	w22-24					***		
	8N	0.435 ± 0.019	0.477 ± 0.017	10.423 ± 2.529	0.783	* **0.014**	1.000	0.121	* **0.021**	w20-22	0.082	*** **<0.001**	0.127	**I** 0.004**
					1.000	* **0.018**	1.000	0.996	0.093	w22-24	0.237	*** **<0.001**		
**PTH (0N: n=10; 8N: n=9)**														
min_R	0N	0.257 ± 0.006	0.264 ± 0.007	3.684 ± 5.304	0.997	0.579	1.000	w20-22	0.993	0.968	0.090	0.497	-	-
					1.000	0.622	0.860	w22-24	0.792	1.000	0.658	*** **<0.001**		
	8N	0.212 ± 0.019	0.241 ± 0.014	34.784 ± 30.913	** 0.002	0.738	0.183	0.090	0.468	0.629	w20-22	0.994	0.277	0.578
					0.625	1.000	1.000	0.658	1.000	0.542	w22-24	0.159		
a	0N	0.245 ± 0.014	0.231 ± 0.016	-0.844 ± 11.363	0.596	0.928	0.505	w20-22	0.969	0.673	0.682	* **0.039**	-	-
					0.988	0.105	1.000	w22-24	0.294	0.998	0.437	*** **<0.001**		
	8N	0.296 ± 0.017	0.297 ± 0.027	0.065 ± 8.468	** 0.006	0.988	* **0.012**	0.682	0.998	* **0.025**	w20-22	0.809	0.490	0.381
					* 0.029	1.000	0.577	0.437	1.000	0.154	w22-24	* **0.043**		
b	0N	0.137 ± 0.012	0.121 ± 0.007	-2.950 ± 14.099	1.000	1.000	0.965	w20-22	1.000	0.834	0.778	0.454	-	-
					0.884	0.491	1.000	w22-24	0.714	0.830	1.000	0.966		
	8N	0.110 ± 0.017	0.112 ± 0.010	43.069 ± 41.462	0.332	0.878	1.000	0.778	0.554	1.000	w20-22	* **0.017**	0.765	0.326
					0.667	0.269	1.000	1.000	0.498	0.632	w22-24	0.864		
y0+a	0N	0.502 ± 0.013	0.495 ± 0.011	-0.762 ± 3.604	0.639	1.000	0.189	w20-22	0.995	0.121	1.000	0.172	-	-
					0.898	0.130	0.999	w22-24	0.348	0.996	0.630	*** **<0.001**		
	8N	0.508 ± 0.017	0.538 ± 0.017	6.339 ± 3.056	0.495	1.000	0.131	1.000	1.000	0.082	w20-22	0.302	0.273	0.218
					* 0.024	1.000	0.325	0.630	1.000	0.237	w22-24	0.128		

**SclAB (0N: n=9; 8N: n=8)**														
min_R	0N	0.245 ± 0.005	0.244 ± 0.011	-0.269 ± 4.322	0.740	0.989	0.999	0.993	w20-22	1.000	0.468	0.932	-	-
					0.786	1.000	1.000	0.792	w22-24	0.684	1.000	0.099		
	8N	0.225 ± 0.014	0.204 ± 0.016	-7.877 ± 6.875	0.070	0.999	0.682	0.497	0.932	0.979	0.994	w20-22	0.717	0.074
					*** <0.001	* 0.016	0.071	*** <0.001	0.099	*** <0.001	0.159	w22-24		
a	0N	0.276 ± 0.018	0.305 ± 0.034	8.845 ± 8.983	0.067	1.000	0.078	0.969	w20-22	0.141	0.998	0.400	-	-
					* 0.012	1.000	0.421	0.294	w22-24	0.088	1.000	0.079		
	8N	0.343 ± 0.036	0.404 ± 0.021	29.009 ± 17.894	*** <0.001	0.159	*** <0.001	* 0.039	0.400	*** <0.001	0.809	w20-22	0.512	0.839
					*** <0.001	* 0.013	*** <0.001	*** <0.001	0.079	*** <0.001	* 0.043	w22-24		
b	0N	0.144 ± 0.008	0.158 ± 0.015	10.653 ± 10.903	1.000	0.978	0.848	1.000	w20-22	0.624	0.554	0.734	-	-
					0.997	1.000	0.761	0.714	w22-24	1.000	0.498	0.999		
	8N	0.175 ± 0.011	0.145 ± 0.019	-14.499 ± 11.321	0.491	0.088	0.065	0.454	0.734	* 0.023	* 0.017	w20-22	0.184	0.311
					1.000	0.999	0.975	0.966	0.999	1.000	0.864	w22-24		
y0+a	0N	0.520 ± 0.015	0.549 ± 0.027	5.211 ± 3.311	0.174	0.998	* 0.037	0.995	w20-22	* 0.021	1.000	0.604	-	-
					** 0.005	1.000	0.139	0.348	w22-24	0.093	1.000	0.299		
	8N	0.568 ± 0.026	0.609 ± 0.014	9.076 ± 6.230	*** <0.001	0.110	*** <0.001	0.172	0.604	*** <0.001	0.302	w20-22	0.854	0.502
					*** <0.001	0.107	*** <0.001	*** <0.001	0.299	*** <0.001	0.128	w22-24		

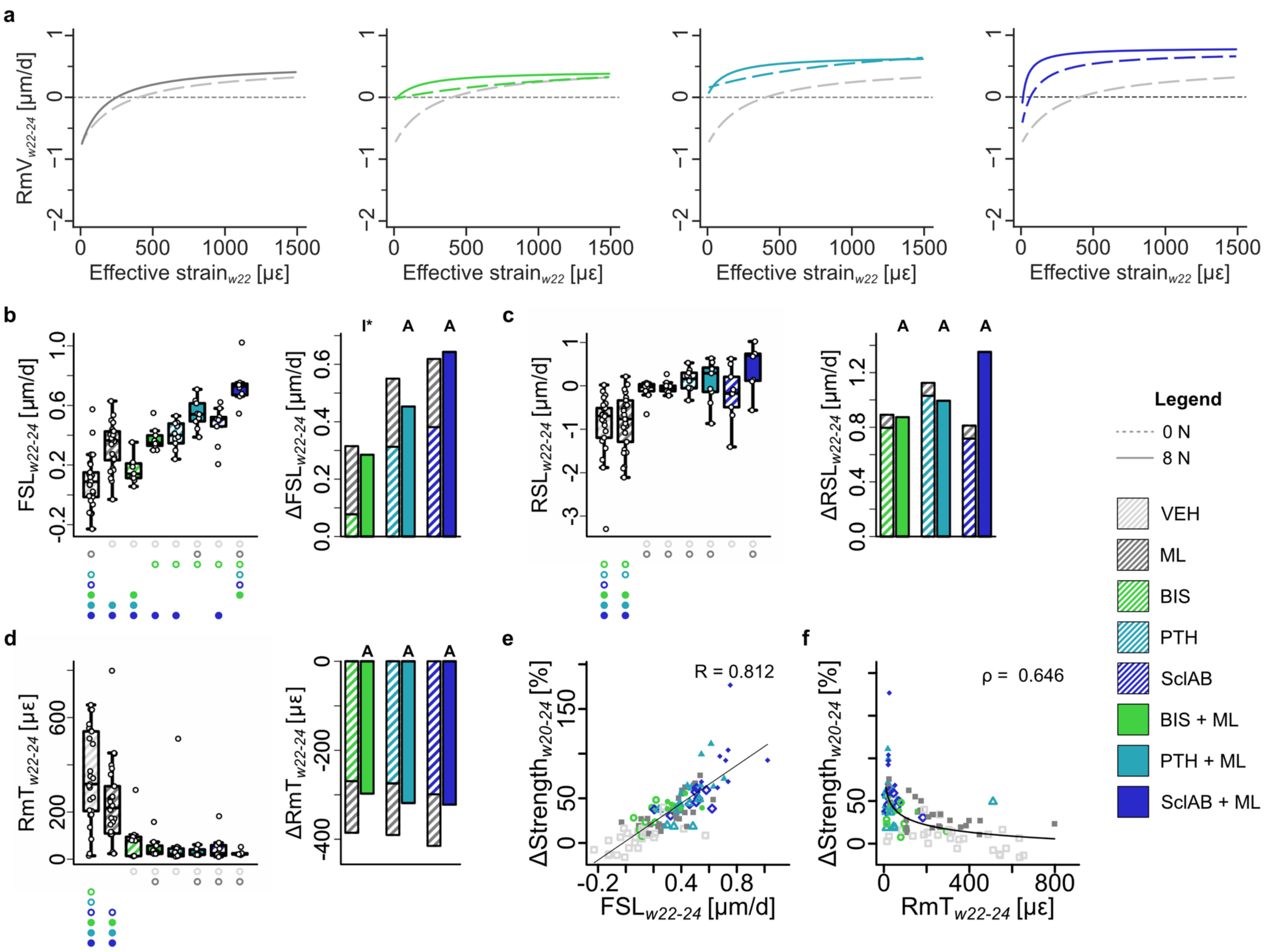
a
RmV w22-24 [µm/d]
Effective strain w22 [µε]
b
FSL w22-24 [µm/d]
ΔFSL w22-24 [µm/d]
I*
A
c
RSL w22-24 [µm/d]
ΔRSL w22-24 [µm/d]
d
RmT w22-24 [µε]
ΔRmT w22-24 [µε]
e
ΔStrength w20-24 [%]
FSL w22-24 [µm/d]
R = 0.812
f
ΔStrength w20-24 [%]
RmT w22-24 [µε]
ρ = 0.646
Legend
0 N
8 N
VEH
ML
BIS
PTH
SclAB
BIS + ML
PTH + ML
SclAB + ML

Supplementary Figure S.4: (Re)modeling velocity curves in mono- and combined treatments as an approximation of the mechanostat theory. (a) (re)modeling velocity (RmV) curve fits (taking all animals per group into account) for VEH / ML, BIS / BIS + ML, PTH / PTH + ML, SclAB / SclAB + ML; **(b)** formation saturation level (FSL) **(c)** resorption saturation level (RSL), **(d)** (re)modeling threshold (RmT) **(b-d)** Each data point represents one animal (biological replicate; VEH: n=25, ML: n=26, BIS 0N: n=9, BIS 8N: n=9, PTH 0N: n=10, PTH 8N: n=9, SclAB 0N: n=9, SclAB 8N: n=8). The box spans the interquartile range (IQR) from the 25th to the 75th percentile. The median is shown as a line inside the box. Whiskers extend to the most extreme data points within 1.5 × IQR, points beyond this range are plotted as outliers. Δ denotes difference from VEH as no baseline value from week 20 was available. Bars show group means of sum of single vs. combined treatment indicating: antagonistic (I), additive (A) or synergistic (S) effects determined via treatment:loading interaction in a linear model on the absolute values. Significances were calculated using one-way ANOVA followed by Tukey’s post-hoc test; significant differences are depicted with a symbol in the color of the corresponding group (o: to monotreatment, ●: to combined treatment, see Supplementary Table S.1 for color encoding, p<0.05). **(g)** Linear regression of ΔStrength with FSL, and power-law regression with RmT in week interval w22-24. Source data are provided as a Source Data file.

Supplementary Dataset S.9: Mechanostat parameters were derived from hyperbolic remodeling velocity curves as described by Marques et al.[3]. Formation (FSL) and resorption (RSL) saturation level curve fits were obtained by fitting each animal individually (see Statistical Methods section). One-way ANOVA in each time interval for the shown animal groups followed by Tukey’s post-hoc test was used on the absolute values. Synergy (S) or antagonism (I) between treatment and mechanical loading was assessed using the interaction term “treatment:loading” from a fitted linear model applied to the absolute values within each interval, because FSL and RSL can obtain negative values. ‘w20-22’ or ‘w22-24’ denotes the reference group; asterisks (*, **, ***) indicate groups that are significantly different from it. Significance is indicated as: *p<0.05, **p<0.01, ***p<0.001.

Readout	N	w20-22	w22-24	%-change between w20-22 and w22-24 [%]	o	o	o	o	o	•	•	•	Synergy w20-22	Synergy w22-24
**VEH (0N: n=25; 8N: n=26)**														
FSL	0N	-0.110 ± 0.030	0.089 ± 0.035	-179.946 ± 55.587	w20-22	*** <0.001	*** <0.001	*** <0.001	*** <0.001	*** <0.001	*** <0.001	*** <0.001	-	-
					w22-24	*** <0.001	0.836	*** <0.001	*** <0.001	*** <0.001	*** <0.001	*** <0.001		
	8N	0.268 ± 0.039	0.326 ± 0.030	39.760 ± 246.153	*** <0.001	w20-22	0.854	0.828	0.999	0.918	*** <0.001	*** <0.001	-	-
					*** <0.001	w22-24	0.067	0.826	0.138	0.968	** 0.003	*** <0.001		
RSL	0N	-1.564 ± 0.183	-0.905 ± 0.143	-13.991 ± 21.592	w20-22	0.134	*** <0.001	0.326	0.373	*** <0.001	*** <0.001	* 0.010	-	-
					w22-24	0.999	** 0.010	*** <0.001	* 0.03	** 0.003	*** <0.001	*** <0.001		
	8N	-0.971 ± 0.200	-0.810 ± 0.126	39.272 ± 32.069	0.134	w20-22	0.096	1.000	1.000	* 0.046	*** <0.001	0.633	-	-
					0.999	w22-24	* 0.035	*** <0.001	0.092	* 0.012	** 0.002	*** <0.001		
RmT	0N	593.596 ± 20.800	347.749 ± 39.075	-37.136 ± 8.389	w20-22	0.158	*** <0.001	** 0.002	* 0.011	*** <0.001	*** <0.001	*** <0.001	-	-
					w22-24	0.084	*** <0.001	*** <0.001	*** <0.001	*** <0.001	*** <0.001	*** <0.001		
	8N	441.163 ± 59.165	231.863 ± 33.763	-32.738 ± 11.718	0.158	w20-22	0.138	0.373	0.675	*** <0.001	* 0.039	** 0.003	-	-

					0.084	w22-24	0.112	0.068	* 0.027	* 0.029	** 0.009	* 0.012		
**BIS (0N: n=9; 8N: n=9)**														
FSL	0N	0.175 ± 0.051	0.166 ± 0.029	-136.028 ± 75.652	*** <0.001	0.854	w20-22	1.000	0.997	0.380	*** <0.001	*** <0.001	-	-
					0.836	0.067	w22-24	** 0.008	*** <0.001	* 0.04	*** <0.001	*** <0.001		
	8N	0.349 ± 0.050	0.374 ± 0.026	471.955 ± 478.813	*** <0.001	0.918	0.380	0.347	0.810	w20-22	0.056	** 0.005	**I* 0.033**	0.715
					*** <0.001	0.986	* 0.04	1.000	0.818	w22-24	0.176	*** <0.001		
RSL	0N	-0.109 ± 0.066	-0.109 ± 0.075	-82.421 ± 97.152	*** <0.001	0.096	w20-22	0.360	0.396	1.000	0.928	0.994	-	-
					** 0.010	* 0.035	w22-24	0.985	1.000	1.000	0.995	0.466		
	8N	-0.023 ± 0.138	-0.031 ± 0.048	111.987 ± 247.604	*** <0.001	* 0.096	0.360	0.396	1.000	w20-22	0.928	0.994	0.245	0.945
					** 0.003	* 0.012	1.000	0.999	0.999	w22-24	1.000	0.657		
RmT	0N	225.688 ± 75.307	78.277 ± 29.808	44.450 ± 71.449	*** <0.001	0.138	w20-22	1.000	0.993	0.749	1.000	0.941	-	-
					*** <0.001	0.112	w22-24	1.000	1.000	1.000	0.996	0.995		
	8N	69.615 ± 26.501	50.264 ± 14.667	13.477 ± 26.845	*** <0.001	*** <0.001	0.749	0.397	0.254	w20-22	0.932	1.000	0.971	0.246
					*** <0.001	* 0.029	1.000	1.000	1.000	w22-24	1.000	1.000		
**PTH (0N: n=10; 8N: n=9)**														
FSL	0N	0.176 ± 0.045	0.401 ± 0.029	123.157 ± 90.690	*** <0.001	0.828	1.000	w20-22	0.997	0.347	*** <0.001	*** <0.001	-	-
					*** <0.001	0.826	** 0.008	w22-24	0.959	1.000	0.354	*** <0.001		
	8N	0.594 ± 0.062	0.542 ± 0.033	-3.173 ± 8.406	*** <0.001	*** <0.001	*** <0.001	*** <0.001	*** <0.001	0.056	w20-22	0.985	0.641	0.207
					*** <0.001	** 0.003	*** <0.001	0.354	0.955	0.176	w22-24	0.101		
RSL	0N	-0.902 ± 0.189	0.125 ± 0.080	-140.371 ± 26.161	0.326	1.000	0.360	w20-22	1.000	0.233	* 0.019	0.884	-	-
					*** <0.001	*** <0.001	0.985	w22-24	0.927	0.999	1.000	0.928		
	8N	0.336 ± 0.192	0.089 ± 0.165	162.411 ± 219.057	*** <0.001	*** <0.001	0.928	* 0.019	* 0.025	0.977	w20-22	0.519	0.131	0.656
					*** <0.001	** 0.002	0.995	1.000	0.966	1.000	w22-24	0.893		
RmT	0N	273.237 ± 43.684	73.302 ± 48.799	-69.398 ± 20.353	** 0.002	0.373	1.000	w20-22	1.000	0.397	0.984	0.704	-	-
					*** <0.001	0.068	1.000	w22-24	1.000	1.000	0.997	0.997		
	8N	186.112 ± 99.819	29.098 ± 5.321	-32.602 ± 23.431	*** <0.001	* 0.039	1.000	0.984	0.929	0.932	w20-22	0.994	0.564	0.330
					*** <0.001	** 0.009	0.996	0.997	1.000	1.000	w22-24	1.000		
**SclAB (0N: n=9; 8N: n=8)**														
FSL	0N	0.230 ± 0.048	0.470 ± 0.043	-141.051 ± 252.882	*** <0.001	0.999	0.997	0.997	w20-22	0.810	*** <0.001	*** <0.001	-	-
					*** <0.001	0.138	*** <0.001	0.959	w22-24	0.818	0.955	** 0.004		
	8N	0.669 ± 0.071	0.732 ± 0.048	16.268 ± 10.997	*** <0.001	*** <0.001	*** <0.001	*** <0.001	*** <0.001	** 0.005	0.985	w20-22	0.508	0.734
					*** <0.001	*** <0.001	*** <0.001	*** <0.001	** 0.004	*** <0.001	0.101	w22-24		

RSL	0N	-0.900 ± 0.112	-0.188 ± 0.208	-62.023 ± 31.429	0.373	1.000	0.396	1.000	w20-22	0.264	* 0.025	0.898	-	-
					* 0.03	0.092	1.000	0.927	w22-24	0.999	0.966	0.293		
	8N	-0.406 ± 0.296	0.446 ± 0.182	-36.460 ± 82.623	* 0.01	0.633	0.994	0.884	0.898	0.972	0.519	w20-22	0.824	0.095
					*** <0.001	*** <0.001	0.466	0.928	0.293	0.657	0.893	w22-24		
RmT	0N	303.569 ± 56.004	48.737 ± 18.175	-84.419 ± 3.720	* 0.011	0.675	0.993	1.000	w20-22	0.254	0.929	0.530	-	-
					*** <0.001	* 0.027	1.000	1.000	w22-24	1.000	1.000	1.000		
	8N	108.929 ± 28.828	25.493 ± 3.914	-64.168 ± 8.188	*** <0.001	** 0.003	0.941	0.704	0.530	1.000	0.994	w20-22	0.716	0.233
					*** <0.001	* 0.012	0.995	0.997	1.000	1.000	1.000	w22-24		

References

1. Lambers FM, Schulte FA, Kuhn G, Webster DJ, Muller R. Mouse tail vertebrae adapt to cyclic mechanical loading by increasing bone formation rate and decreasing bone resorption rate as shown by time-lapsed in vivo imaging of dynamic bone morphometry. *Bone* **49**, 1340-1350 (2011).

2. Schulte FA*, et al.* Local mechanical stimuli regulate bone formation and resorption in mice at the tissue level. *PLoS One* **8**, e62172 (2013).

3. Marques FC, Boaretti D, Walle M, Scheuren AC, Schulte FA, Müller R. Mechanostat parameters estimated from time-lapsed micro-computed tomography data of mechanically driven bone adaptation are logarithmically dependent on loading frequency. *Front Bioeng Biotech* **11**, (2023).
