# Supplementary material for: Combined physical and pharmacological anabolic osteoporosis therapies increase bone response and mechanoregulation in female mice
Source: Nat Commun. 2026 Mar 10;17:3759. doi: 10.1038/s41467-026-70309-2 (PMC13106859; doi:10.1038/s41467-026-70309-2)
Supplement: Supplementary file 2 — Description of Additional Supplementary Files [file 41467_2026_70309_MOESM2_ESM.pdf]

<u>Description of Additional Supplementary Files</u>

Supplementary Dataset S.1: Color encoding for significant differences to a specific group. Significances are depicted with a symbol in the color of the corresponding group using two-way ANOVA for the shown groups followed by Tukey's post-hoc test ($p<0.05$: o: to monotreatment, ●: to combined treatment, ooooo●●●: to VEH 0N, to VEH 8N, to BIS 0N, to PTH 0N, to ASC 0N, to BIS 8N, to PTH 8N, to ASC 8N).

Supplementary Dataset S.2: Static morphometric and mechanical parameters of the trabecular compartment of non-loaded (0N) and loaded (8N) 6th caudal vertebrae at weeks 15, 20, 22, and 24 (w15, w20, w22, w24). Values are presented as group means ± standard error, Δ represents the %-change from week 20 to 24 (Δw20-24). Two-way ANOVA over time for the shown groups followed by Tukey's post-hoc test was used on Δw20-24. 'x' denotes the reference group; asterisks (*, **, ***) indicate groups that are significantly different from 'x'. Significance is indicated as: *$p<0.05$, **$p<0.01$, ***$p<0.001$. Synergy (S) or antagonism (I) between treatment and mechanical loading was assessed using the interaction term "treatment:loading" from a fitted linear model applied to Δw20-24.

Supplementary Dataset S.3: Absolute values and %-changes in bone volume fraction (BV/TV) of the three vehicle groups of the four experiments (see Methods). The animals in Experiment 3 (SclAB) were supplied by a different provider; see Methods section "Study design". The VEH 0N groups received sham loading, the VEH 8N groups cyclic mechanical loading according to an established loading protocol1 in the sixth caudal vertebra between week 20 and 24. Values are presented as group means ± standard error in weeks 15, 20, 22, and 24 (w15, w20, w22, w24), Δ represents the %-change from week 20 to 24 (Δw20-24). One-way ANOVA for the shown vehicle groups followed by Tukey's post-hoc test was used to calculate significant differences between groups. *$p<0.05$, **$p<0.01$, ***$p<0.001$.

Supplementary Dataset S.4: Cortical morphometric and full mechanical parameters of the non-loaded (0N) and loaded (8N) 6th caudal vertebrae. Values are given as group means ± standard error, Δ represents the %-change from week 20 to 24 (Δw20-24). 'full' denotes that both trabecular and cortical compartments were included. Two-way ANOVA over time for the shown animal groups followed by Tukey's post-hoc test was used on Δw20-24. 'x' denotes the reference group; asterisks (*, **, ***) indicate groups that are significantly different from 'x'. Significance is indicated as: *$p<0.05$, **$p<0.01$, ***$p<0.001$. Synergy (S) or antagonism (I) between treatment and mechanical loading was assessed using the interaction term "treatment:loading" from a fitted linear model applied to Δw20-24.

Supplementary Dataset S.5: Dynamic morphometric parameters of the non-loaded (0N) and loaded (8N) trabecular compartments of 6th caudal vertebrae, given as group means ± standard error in week intervals w20-22 and w22-24. The %-change between week intervals w20-22 and w22-24, calculated per animal, is depicted as well. One-way ANOVA in each time interval for the shown animal groups followed by Tukey's post-hoc test was used on the absolute values. 'w20-22' or 'w22-24' denotes the reference group; asterisks (*, **, ***) indicate groups that are significantly different from it. Significance is indicated as: *$p<0.05$, **$p<0.01$, ***$p<0.001$. Synergy (S) or antagonism (I) between treatment and

mechanical loading was assessed using the interaction term "treatment:loading" from a fitted linear model applied to the log2-transformed absolute values within each interval.

Supplementary Dataset S.6: Group means ± standard error of the absolute strain energy density (SED) values in formation (F), quiescence (Q) and resorption (R) sites are reported for all vehicle and treatment groups for both time intervals. Furthermore, mean SED at formation, quiescent and resorption sites expressed as percent difference from the mean (over all animals) quiescent SED are reported as |F-Q| and |Q-R|. Normalization to the mean (over all animals) quiescent SED preserves group properties thus allows statistical comparison of relative differences between F,Q,R regions. P-values were calculated using pairwise two-sided Wilcoxon signed-rank tests with Bonferroni correction. Significance is indicated as: *p<0.05, **p<0.01, ***p<0.001.

Supplementary Dataset S.7: Saturating exponential curve fits for formation slope values were conducted by fitting the function $f(x)=y_0+a \cdot (1-\exp(-b \cdot x))$ to the first 40% of the normalized SED/SEDmax interval for each animal individually. Coefficients are presented as group means ± standard error. Parameter y_0 reflects the level of formation in the absence of mechanical stimulus (i.e. at SED = 0), representing non-targeted formation. Coefficient a indicates the maximal increase in formation in response to SED, while y_0+a defines the plateau, or the maximum potential formation probability at high SED. b represents the sensitivity or rate of response, i.e. higher b-values indicate that the curve rises more quickly, reaching the plateau earlier (i.e. formation increases rapidly with even low SED). Synergy (S) or antagonism (I) between treatment and mechanical loading was assessed using the interaction term "treatment:loading" from a fitted linear model applied to the log2-transformed absolute values within each interval. P-values were obtained using one-way ANOVA in each time interval for the shown groups followed by Tukey's post-hoc test to account for multiple comparisons. 'w20-22' or 'w22-24' denotes the reference group; asterisks (*, **, ***) indicate groups that are significantly different from it. Significance is indicated as: *p<0.05, **p<0.01, ***p<0.001.

Supplementary Dataset S.8: Decaying exponential curve fits for resorption were conducted by fitting the function $f(x)=y_0+a \cdot \exp(-b \cdot x)$ to the first 40% of the normalized SED/SEDmax interval for each animal individually. The fitted coefficients for each animal are presented as group means ± standard error. Resorption asymptote minR represents the saturation level of resorption at high mechanical signal, i.e. the minimum resorption probability ($x \to \infty$), indicative of the amount of non-targeted resorption. Coefficient a represents the magnitude of the response, i.e. the difference between the initial and the minimum resorption probability. $a+y_0$ represents the peak resorption probability at low SED. Coefficient b is the rate of decay, representing how quickly the probability of resorption drops as SED increases. Synergy (S) or antagonism (I) between treatment and mechanical loading was assessed using the interaction term "treatment:loading" from a fitted linear model applied to the log2-transformed absolute values within each interval. P-values were obtained using one-way ANOVA in each time interval for the shown groups followed by Tukey's post-hoc test to account for multiple comparisons. 'w20-22' or 'w22-24' denotes the reference group; asterisks (*, **, ***) indicate groups that are significantly different from it. Significance is indicated as: *p<0.05, **p<0.01, ***p<0.001.

Supplementary Dataset S.9: Mechanostat parameters were derived from hyperbolic remodeling velocity curves as described by Marques et al.3. Formation (FSL) and resorption (RSL) saturation level curve fits were obtained by fitting each animal individually (see Statistical Methods section). One-way ANOVA in each time interval for the shown animal groups followed by Tukey's post-hoc test was used on the absolute values. Synergy (S) or antagonism (I) between treatment and mechanical loading was assessed using the interaction term "treatment:loading" from a fitted linear model applied to the absolute values within each interval, because FSL and RSL can obtain negative values. 'w20-22' or 'w22-24' denotes the reference group; asterisks (*, **, ***) indicate groups that are significantly different from it. Significance is indicated as: *p<0.05, **p<0.01, ***p<0.001.
